# Supplementary material for: Effects of a modified muscle sparing posterior technique in hip hemiarthroplasty for displaced intracapsular fractures on postoperative function compared to a standard lateral approach (HemiSPAIRE): protocol for a randomised controlled trial
Source: BMJ Open. 2021 Jun 8;11(6):e045652. doi: 10.1136/bmjopen-2020-045652 (PMC8190059; doi:10.1136/bmjopen-2020-045652)
Supplement: Supplementary data [file bmjopen-2020-045652supp001.pdf]

Participant consent form

The HemiSPAIRE study:
The effects of a modified muscle sparing posterior technique (SPAIRE) in hip hemiarthroplasty for displaced intracapsular fractures on post-operative function compared to a standard lateral approach; a randomised controlled trial.

CONSENT FORM

Please see that the consent form is in two parts, you do not have to sign both parts:

Part 1 on Page 1:
This is the main consent for your general participation in the study and if you agree to taking part.

Part 2 on Page 2 is optional; you can choose if you wish to take part.
This is about whether you would agree to being interviewed about your experiences of recovery from surgery. It also includes a similar section about data collection.

PART 1: MAIN STUDY CONSENT FORM

Site Details:

	Please initial box
1. I confirm that I have read and understand the information sheet dated version for the above study and have had the opportunity to consider the information, ask questions and have had these answered satisfactorily.	
2. I agree to my usual care giver being informed of my participation in this study and updated with information from this study relevant to my medical care.	
3. I understand that my participation is voluntary and that I am free to withdraw at any time, without giving any reason and without my medical care or legal rights being affected.	
4. I understand that relevant sections of my medical notes and data collected during the study may be looked at by individuals, from regulatory authorities or from the NHS Trust, where it is relevant to my taking part in the research. I give permission for these individuals to have access to my records.	
5. I understand that identifiable data already collected as part of the research study can be retained for up to 5 years, even if I decide to withdraw from the study and that it will only be used for this study.	
6. I understand that the anonymised data collected from the study will be retained in secure storage during the study and after the study will be stored in a repository.	
7. I agree to my General Practitioner being informed of my participation in the study.	
8. I agree to take part in the above study.	

When you have initialled the boxes above, please complete below including the date yourself.

Name of Participant *(BLOCK CAPITALS)* **Date** **Signature**

I have explained the study to the above patient and he/she has indicated his/her willingness to take part in the study.

Name of Researcher *(BLOCK CAPITALS)* **Date** **Signature**

You will be given a copy of this consent form and the information sheet to keep. A copy of the consent form will be filed in your patient notes, one will be filed with the study records and one may be sent to the Research Sponsor (Royal Devon and Exeter).

Price A, *et al. BMJ Open* 2021; 11:e045652. doi: 10.1136/bmjopen-2020-045652

Participant consent form

The HemiSPAIRE study:
The effects of a modified muscle sparing posterior technique (SPAIRE) in hip hemiarthroplasty for displaced intracapsular fractures on post-operative function compared to a standard lateral approach; a randomised controlled trial.

The HemiSPAIRE study: The effects of a modified muscle sparing posterior technique (SPAIRE) in hip hemiarthroplasty for displaced intracapsular fractures on post-operative function compared to a standard lateral approach; a randomised controlled trial.

PART 2: OPTIONAL CONSENT FORM

Part 2: This section is optional; you can choose if you wish to take part or not and it will not affect your participation in the main part of the study.

This consent form is about whether you would agree to:

- *Being interviewed about your experiences of recovery from surgery?*

Please only initial the boxes that you wish to consent to, thank you.

Site Details

	**Please only initial the boxes that apply**
1. I am willing to be interviewed about my experiences of recovery from hip fracture surgery and for this interview to be audio recorded for research purposes	
2. I agree to the data collected for this additional part of the above study being retained for up to 5 years.	
3. I agree to my data from this study being shared with other health researchers after my personal identifying information has been removed. I understand that it will only be used towards improving health outcomes by assessing the types of treatment that I have agreed to participate in for the main study.	
4. I understand that quotes from my interview may be used in study publications such as journal articles, and that these quotes will not contain any information that would enable someone to identify me.	
5. I agree to this additional part of the above study	

Name of Participant *(BLOCK CAPITALS)* **Date** **Signature**

I have explained the additional part of study to the above patient and he/she has indicated which parts apply.

Name of Researcher *(BLOCK CAPITALS)* **Date** **Signature**

Price A, *et al. BMJ Open* 2021; 11:e045652. doi: 10.1136/bmjopen-2020-045652

Consultee declaration form

National Institute for Health Research

The HemiSPAIRE study:
The effects of a modified muscle sparing posterior technique (SPAIRE) in hip hemiarthroplasty for displaced intracapsular fractures on post-operative function compared to a standard lateral approach; a randomised controlled trial.

CONSULTEE DECLARATION FORM

Site Details:

	Please initial box
1. I [*name of consultee (see below)]* have been consulted about [*name of potential participant* ______________ ______________*]*'s participation in this research project. I have had the opportunity to ask questions about the study and understand what is involved.	
2. In my opinion he/she would have no objection to taking part in the above study.	
3. I understand that his/her participation is voluntary and that they are free to withdraw at any time, without giving any reason and without their medical care or legal rights being affected.	
4. I understand that relevant sections of his/her medical notes and data collected during the study may be looked at by individuals from the HemiSPAIRE project team, and from regulatory authorities where it is relevant to his/her taking part in the research. I give permission for these individuals to have access to their records.	
5. I understand that the information collected about him/her may be published and shared anonymously with other researchers. The information may be used to support other research in the future.	
6. I understand that the anonymised data collected from the study will be retained in secure storage during the study and after the study will be stored in a repository.	
7. I agree to his/her General Practitioner being informed of their participation in the study.	

When you have initialled the boxes above, please complete below including the date yourself.

Name of Consultee *(BLOCK CAPITALS)* **Date** **Signature**

Relationship to Patient *(BLOCK CAPITALS)*

I have explained the study to the above consultee and he/she has indicated his/her willingness to consent to the patient taking part in the study.

Name of Researcher *(BLOCK CAPITALS)* **Date** **Signature**

You will be given a copy of this declaration form and the information sheet to keep. A copy of the declaration form will be filed in the patient notes, one will be filed with the study records and one may be sent to the Research Sponsor (Royal Devon and Exeter).
