# Supplementary material for: Effects of a modified muscle sparing posterior technique in hip hemiarthroplasty for displaced intracapsular fractures on postoperative function compared to a standard lateral approach (HemiSPAIRE): protocol for a randomised controlled trial
Source: BMJ Open. 2021 Jun 8;11(6):e045652. doi: 10.1136/bmjopen-2020-045652 (PMC8190059; doi:10.1136/bmjopen-2020-045652)
Supplement: Author's manuscript [file bmjopen-2020-045652.draft_revisions.pdf]

BMJ Open is committed to open peer review. As part of this commitment we make the peer review history of every article we publish publicly available.

When an article is published we post the peer reviewers' comments and the authors' responses online. We also post the versions of the paper that were used during peer review. These are the versions that the peer review comments apply to.

The versions of the paper that follow are the versions that were submitted during the peer review process. They are not the versions of record or the final published versions. They should not be cited or distributed as the published version of this manuscript.

BMJ Open is an open access journal and the full, final, typeset and author-corrected version of record of the manuscript is available on our site with no access controls, subscription charges or pay-per-view fees (http://bmjopen.bmj.com).

If you have any questions on BMJ Open's open peer review process please email info.bmjopen@bmj.com

BMJ Open

The effects of a modified muscle sparing posterior technique in hip hemiarthroplasty for displaced intracapsular fractures on post-operative function compared to a standard lateral approach (HemiSPAIRE); protocol for a randomised controlled trial.

Journal:	*BMJ Open*
Manuscript ID	bmjopen-2020-045652
Article Type:	Protocol
Date Submitted by the Author:	08-Oct-2020

Complete List of Authors:	Price, Anna; University of Exeter Medical School, Clinical Trials Unit<br>Ball, Susan; University of Exeter Medical School, NIHR CLAHRC South West Peninsula (PenCLAHRC)<br>Rhodes, Shelley; University of Exeter Medical School, Clinical Trials Unit<br>Wickins, Robert; Royal Devon and Exeter NHS Foundation Trust, Physiotherapy<br>Gordon, Elizabeth; Royal Devon and Exeter NHS Foundation Trust, Research<br>Aylward, Alex; University of Exeter Medical School, Public and Patient Involvement Group<br>Cockcroft, Emma; University of Exeter Medical School<br>Morgan-Trimmer, Sarah; University of Exeter Medical School, Psychology Applied to Health (PAtH) Group, Institute of Health Research<br>Powell, Roy; Royal Devon and Exeter NHS Foundation Trust, Research Design Service<br>Timperley, John; Royal Devon and Exeter NHS Foundation Trust, Exeter, Hip Unit, Princess Elizabeth Orthopaedic Centre<br>Charity, John; Royal Devon and Exeter NHS Foundation Trust, Exeter, Hip Unit, Princess Elizabeth Orthopaedic Centre

Keywords:	Hip < ORTHOPAEDIC & TRAUMA SURGERY, SURGERY, ORTHOPAEDIC & TRAUMA SURGERY, Clinical trials < THERAPEUTICS

SCHOLARONE™
Manuscripts

I, the Submitting Author has the right to grant and does grant on behalf of all authors of the Work (as defined in the below author licence), an exclusive licence and/or a non-exclusive licence for contributions from authors who are: i) UK Crown employees; ii) where BMJ has agreed a CC-BY licence shall apply, and/or iii) in accordance with the terms applicable for US Federal Government officers or employees acting as part of their official duties; on a worldwide, perpetual, irrevocable, royalty-free basis to BMJ Publishing Group Ltd ("BMJ") its licensees and where the relevant Journal is co-owned by BMJ to the co-owners of the Journal, to publish the Work in this journal and any other BMJ products and to exploit all rights, as set out in our licence.

The Submitting Author accepts and understands that any supply made under these terms is made by BMJ to the Submitting Author unless you are acting as an employee on behalf of your employer or a postgraduate student of an affiliated institution which is paying any applicable article publishing charge ("APC") for Open Access articles. Where the Submitting Author wishes to make the Work available on an Open Access basis (and intends to pay the relevant APC), the terms of reuse of such Open Access shall be governed by a Creative Commons licence – details of these licences and which Creative Commons licence will apply to this Work are set out in our licence referred to above.

Other than as permitted in any relevant BMJ Author's Self Archiving Policies, I confirm this Work has not been accepted for publication elsewhere, is not being considered for publication elsewhere and does not duplicate material already published. I confirm all authors consent to publication of this Work and authorise the granting of this licence.

For peer review only

TITLE: The effects of a modified muscle sparing posterior technique in hip hemiarthroplasty for displaced intracapsular fractures on post-operative function compared to a standard lateral approach (HemiSPAIRE); protocol for a randomised controlled trial.

Authors

Anna Price[1], Susan Ball[2], Shelley Rhodes[1], Robert Wickins[3], Elizabeth Gordon[3], Alex Aylward[2], Emma Cockcroft[2], Sarah Morgan-Trimmer[1], Roy Powell[1,3], A J Timperley[1,3], John Charity[3].

[1]University of Exeter Medical School, Exeter, EX1 2LU, UK.
[2]NIHR Applied Research Collaboration South West Peninsula, University of Exeter Medical School, Exeter, EX1 2LU, UK.
[3]Royal Devon & Exeter Hospital, Exeter, EX2 5DW, UK.

Corresponding author
Dr Anna Price,
Clinical Trials Unit,
University of Exeter Medical School,
College House
St Luke's Campus,
Exeter,
EX1 2LU,
Email: a.price@exeter.ac.uk
01392 726026

Trial sponsor
Dr Alison Kerridge,
Assistant R&D Manager,
NIHR Clinical Research Network: South West Peninsula
Room F6, Bowmoor House,
Royal Devon & Exeter NHS Foundation Trust, Barrack Road,
Exeter, EX2 5DW
Email: alison.kerridge @nhs.net
01392 403055

Key words: Displaced intracapsular hip fracture, Hip hemiarthroplasty, Surgical approach, Muscle sparing, Function, Clinical Trials.

Protocol Version: 2 Date: 13/05/2020

Word count: 3846

For peer review only - http://bmjopen.bmj.com/site/about/guidelines.xhtml

ABSTRACT

Introduction: Currently National Institute for Health and Care Excellence clinical guidelines in the United Kingdom suggest that surgeons performing partial hip replacements (hemiarthroplasty) should consider using the lateral approach. Alternatively, a newer, modified posterior approach using a muscle sparing technique named 'Save Piriformis and Internus, Repairing Externus' (SPAIRE) can be used leaving the major muscles intact. This randomised controlled trial (RCT) aims to compare the SPAIRE approach to the standard lateral approach, to determine if it allows patients to mobilise better and experience improved function after surgery.

Methods and analysis: HemiSPAIRE is a two-arm, assessor-blinded, definitive pragmatic RCT with nested pilot and qualitative studies. Two-hundred and twenty eight participants with displaced intracapsular fractures requiring hip hemiarthroplasty will be individually randomised 1:1 to either the SPAIRE, or control (standard lateral approach) surgical procedure. Outcomes will be assessed at post-operative day three (POD3), and one-hundred and twenty (POD120). The primary outcome measure will be level of function and mobility using the Oxford Hip Score (OHS) at POD120. Secondary outcomes include: De Morton Mobility Index (DEMMI), Cumulated Ambulatory Score (CAS) and Numeric Pain Rating Scale (NPRS) at POD3; DEMMI, NPRS and EQ-5D-5L at POD120, complications, acute and total length of hospital stay, and mortality. Primary analysis will be on an intention-to-treat basis. Participant experiences of the impact of surgery and recovery period will be examined via up to twenty semi-structured telephone interviews.

Ethics and dissemination: The protocol has been approved by Yorkshire and the Humber – Bradford Leeds Research Ethics Committee. Recruitment commenced in November 2019. Findings will be disseminated via research articles in peer-reviewed journals, presentations at conferences, public involvement events, patient groups, and media releases. A summary of the trial findings will be shared with participants at the end of the study.

Registration: ClinicalTrials.gov: trial number NCT04095611.

For peer review only

ARTICLE SUMMARY

Strengths and limitations of this study

- Comprehensive Patient and Public Involvement: outcome measures were inspired by patients who said that mobility and speed to regaining independence are the most important outcomes after partial hip replacement surgery
- Addresses the current evidence gap on the impact on patient mobilisation and function after surgery using different techniques (SPAIRE vs. lateral)
- A pragmatic multicentre study across six NHS Trusts in the South West of England with broad inclusion criteria to recruit a population that will mirror as close as possible this frail group of patients as reduced cognition is not an exclusion criterion
- Due to Covid19, the primary outcome measure was changed to a self-report measure meaning some outcomes were not collected in the manner originally planned

For peer review only

INTRODUCTION

Hip fractures are common in the elderly. Over 20,000 cases of hip hemiarthroplasty (replacement of the fractured femoral head) are performed annually in England, Wales and Northern Ireland (1). Hip fracture patients endure debilitating loss of function, and recovery is often complex and challenging (2). The average total length of stay for hip fracture admissions is over 21 days, representing over 4000 National Health Service (NHS) hospital beds occupied by hip fracture patients at any one time (3).

The National Institute for Health and Care Excellence (NICE) recommends hemiarthroplasty of the hip for the treatment of displaced intracapsular fractures when patients are not eligible for total hip replacement (4). As a commonly performed procedure, it is important to consider innovative hemiarthroplasty techniques that may allow better and safer rehabilitation in this frail group of patients.

When planning a hip hemiarthroplasty, surgeons have a choice of surgical approaches. NICE guidelines (4) currently recommend using a lateral rather than a conventional posterior approach. This advice is based on evidence described as being of “very poor quality” (4). For adequate exposure, the lateral approach requires division and subsequent repair of 50% or more of the tendon attachments of the gluteus medius and minimus muscles on to the greater trochanter. These muscles are essential for normal gait. Literature quoted suggests a reduced dislocation rate for the lateral approach; however this surgery has the disadvantage that the relatively extensive division of tendon attachments required may result in reduced levels of function postoperatively. Recent evidence from a cohort of over 20,000 patients from the Norwegian Hip Fracture Register (5) reported better patient-related outcome measures (pain, patient satisfaction and health-related quality of life) with a standard posterior approach compared to the lateral approach. However, the study also confirmed the higher dislocation rates in the conventional posterior approach group. Some studies report dislocation in up to 10% of patients undergoing this hip procedure through a standard posterior approach and such complications can lead to potentially catastrophic consequences (6).

To address the issue of dislocation in hip arthroplasty, modified surgical procedures have been attempted using muscle sparing techniques. In 2012, Han et al. (6) described a modified posterior approach for use in patients with neurological disorders requiring hip hemiarthroplasty, where the piriformis, gemellus superior, obturator internus and part of quadratus femoris muscles were left intact, which combined with a standard capsule repair led to a reduced incidence of dislocation (6).

In 2016, the Hip Unit at the Royal Devon and Exeter NHS Foundation Trust developed a modified technique using a posterior approach for hip hemiarthroplasties applicable to all

For peer review only - http://bmjopen.bmj.com/site/about/guidelines.xhtml

patients. This approach involves division of only the obturator externus tendon and part of quadratus femoris muscle from their femoral insertions. These are repaired, along with the posterior capsule, at the end of the surgery with strong non-absorbable suture through an enhanced trans-osseous technique onto the posterior aspect of the greater trochanter. The tendon insertions of piriformis, gemellus superior, obturator internus and gemellus inferior muscles are spared, and the abductor muscle insertions are left undisturbed, minimising the potential negative impact of dividing muscles during surgery on post-operative recovery and mobility. This approach, named "SPAIRE", allows the surgeon to "Save Piriformis and Internus, Repairing Externus". These muscles have been shown to act as the main extensor and abductor of the flexed hip which is of significant importance for movements such as rising from a chair or climbing stairs (7). This contrasts to the standard lateral approach, where a significant proportion of the gluteal muscle insertions is divided, potentially impacting hip function. The combination of this muscle sparing approach with an enhanced capsule repair aims to provide sufficient stability to enable patients to mobilise full-weight bearing, without any of the specific restrictions currently included in routine post-operative posterior approach protocols. The preservation of these muscle insertions may replicate or even surpass the improved patient-related outcome measures observed in the study by Kristensen et al. (5) and might achieve other benefits relating to more complete rehabilitation, reduced hospital stay, and diminished requirement for social service support on discharge, with consequent savings from the health and social care budget.

Review of existing evidence

A scoping review identified thirteen studies that compared the traditional posterior approach with the lateral approach, including two randomised controlled trials (RCTs) (8). These evidence a trend for increased incidence of dislocation using the traditional posterior approach versus other approaches, with the exception of one paper describing good results with a modified version of the posterior approach (9). However, this approach was only attempted in a small subset of the population prone to these fractures. Evidence with regard to other outcomes is inconsistent and of limited quality, with few RCTs to inform guidelines(8).

Aims

HemiSPAIRE is a RCT which, with its focus on post-operative function and mobility, aims to provide high quality evidence on the relative benefits of the SPAIRE surgical technique when compared with standard lateral technique. The findings may help to inform and update current guidelines (4). In doing so it aims to contribute to improved function, mobility, and quality of life outcomes for hip fracture patients, many of whom are elderly and frail, and reduce length of hospital stays.

Primary objective

For peer review only - http://bmjopen.bmj.com/site/about/guidelines.xhtml

To test whether the SPAIRE technique improves post-operative function and mobility at 120 days following surgery in adults with a displaced intracapsular hip fracture requiring hemiarthroplasty, compared to the standard lateral approach through conducting a definitive two-arm RCT.

Secondary objectives

Test whether the SPAIRE technique results in improved early function, mobility, pain and quality of life at 120 days, with reduced length of hospital stay, complication rates, and mortality compared to the standard lateral approach, through collecting secondary outcome measures in the trial.

Investigate how patients experience the recovery period and mechanisms of recovery after surgery, by conducting a qualitative study with a sub-sample of patients in each trial arm.

Engage the contribution of a PPI group to ensure the conduct and outputs of the study are relevant and useful to patients.

METHODS AND ANALYSIS

Trial design

This is a definitive pragmatic, multi-centre RCT in patients attending hospital with a displaced intracapsular fracture requiring hip hemiarthroplasty. Patients will be randomised to have their operation performed either by posterior approach (SPAIRE technique) or the standard lateral approach. Patients, ward staff and all research staff involved in post-operative evaluations will remain blinded to allocation. Outcomes will be recorded on the third post-operative day (POD3) and 120 days after surgery (POD120). These include measures of function, mobility, quality of life, pain, surgical complications, hospital length of stay, mortality, discharge destination, and place of residence. Figure 1 shows a flow chart of the trial design.

Trial setting

Six acute hospitals in the South West of England are recruitment centres, with a minimum of two surgeons per site. Training has been provided by the Chief Investigator (CI) and co-applicant AJT, using lectures, mentorship, observations, videos, and one-to-one sessions as necessary. All participating surgeons have carried out a minimum of five cases using the posterior approach (SPAIRE technique) prior to participating in the study, and are prepared to undertake both procedures. Each participating surgeon uses both techniques. If a surgeon is not in equipoise then he/she will not participate.

Participant eligibility criteria

For peer review only - http://bmjopen.bmj.com/site/about/guidelines.xhtml

All patients requiring hemiarthroplasty for a displaced intracapsular hip fracture are considered for inclusion. Inclusion criteria: patients aged 60 years or older presenting with an intracapsular hip fracture requiring hip hemiarthroplasty (4) who are resident in the South West of England. Exclusion criteria: patients who were immobile (unable to walk) before hip fracture, patients not expected to live until POD120 due to chronic illness and receiving surgery for palliative care, and use of femoral stems not of a proven stem design, in line with recommendations set by NICE clinical guideline on hip fracture management (4). A sizeable proportion of this population suffer dementia and/or temporary delirium, and are not excluded; cognitive ability is not part of the eligibility criteria.

Trial procedures

Recruitment and participant identification

When a patient attends hospital with a confirmed intracapsular hip fracture, they are considered for inclusion. If potentially eligible the study is discussed with the patient and/or their carer(s). Surgery usually takes place 12 hours or more after admission or on the day after, providing patients with time to consider their participation. Potential participants are identified by orthopaedic surgeons involved in the study (co-applicants and collaborators) who admit patients under their care.

Consent

Identified patients interested in participating in the study are invited to read the participant information sheet and, if interested, to provide informed consent. Researchers will be present to encourage potential participants to ask questions and to ensure participants fully understand the purpose of the research, risks associated with the intervention, obligations of participation and their right to withdraw at any time. Consent forms must be fully completed and signed before patients are accepted on to the study. If a patient lacks capacity and is unable to consent, potential participation is discussed and consent later sought with a person whose relationship to the patient makes them suitable to act as his/her legal representative e.g. personal or professional consultee. If possible the study is discussed or communicated to the participant in a way appropriate to their understanding. Participants who lack the mental capacity to consent and do not seem in agreement with any part of the study, even if agreement has been given by another, are not included. The participant's general practitioner is notified by letter that his/her patient is participating in the study.

The randomisation scheme

Randomisation is undertaken as late as practically possible. There is no special preparation required in theatre, and no difference in equipment required for either surgical technique. Participants will be individually randomised to receive the SPAIRE or lateral procedure in a 1:1 ratio. Concealed allocation is determined by the UKCRC registered Exeter Clinical Trials Unit (CTU) using a validated password-protected web-based system. Allocation is based on

For peer review only - http://bmjopen.bmj.com/site/about/guidelines.xhtml

random permuted blocks of varying size, and stratified by study site and by cognition level (impaired vs non-impaired). The surgeon is informed of allocation by the CTU via email through nhs.net mail. The CI and Principal Investigator (PI) at site are copied in.

Blinding

Patients, ward staff, and research staff performing outcome assessments are blinded to treatment allocation. Surgeons and operative team are unblinded. An unblinded coordinator transcribes the surgical data to the database. There is no difference between the SPAIRE and lateral approach techniques in the following: surgical time taken, application of surgical dressing or post-operative care. For medicolegal reasons, the surgical approach used in the procedure is specified in the operation notes. A cover sheet is attached in front of the printed operation note stating that the patient is a participant in the trial and reminding the research team to avoid inadvertent unblinding to treatment allocation during post-operative assessments.

Trial treatments

Trial interventions

The two groups differ only in the surgical approach to the hemiarthroplasty. All preparation for surgery, patient positioning, skin incision, other aspects of surgery, surgical dressing and post-operative care, are the same according to current practice.

SPAIRE technique through the posterior approach to the hip

If randomised to this arm of the trial, the SPAIRE technique is performed. This involves a modified muscle-sparing posterior approach where insertions of piriformis, superior gemellus, obturator internus and inferior gemellus are spared with division of only obturator externus and part of quadratus femoris. The single divided tendon and posterior capsule are subsequently repaired with a transosseous repair to their initial position prior to closure. The insertions of the abductor muscles are left intact throughout the procedure.

LATERAL approach to the hip

If randomised to this arm of the trial, the patient is prepared and the operation performed in accordance with criteria set by the study, so as to minimise issues of standardisation with this approach. This means that the gluteus medius and minimus insertions onto the greater trochanter are partially divided anteriorly, leaving the posterior part of their insertions intact. The anterior capsule is divided and subsequently repaired prior to closure, followed by repair of the detached portion of the gluteal muscles.

Trial assessments

For details of trial assessments see table 1.

<u>Table 1: trial assessments, with time-points</u>

For peer review only

For peer review only - http://bmjopen.bmj.com/site/about/guidelines.xhtml

Objective	Outcome Assessment	Tests conducted at:
**Primary**		
Assess postoperative function and mobility.	The Oxford Hip Score (OHS).	120 days after surgery.
**Secondary**		
Assess postoperative function and early mobility.	The Oxford Hip Score (OHS).	3 days after surgery.
	The De Morton Mobility Index (DEMMI) test.	3 and 120 days after surgery.
	The Cumulated Ambulation Score (CAS).	3 days after surgery.
Assess pain.	Numeric pain rating scale (NPRS).	3 and 120 days after surgery.
Measure quality of life.	EuroQol EQ-5D-5L.	120 days after surgery.
Measure hospital stay.	Acute and total length of hospital stay.	120 days after surgery.
Assess complication rates.	Specific hip-related complications: dislocation, nerve injury, periprosthetic fracture, infection within 120 days of operation and need for re-operation (with reasons).	Within 120 days after surgery, recording the date of complication.
Record discharge destination	The percentage of participants who are discharged to their pre-fracture place of residence.	At time of discharge from hospital admission.
Return to place of residence	Place of residence. The percentage of participants returning to their pre-fracture place of residence.	At 120 days after surgery.
Estimate survival.	Mortality within 120 days of the operation.	Within 120 days after surgery, recording the date of death.

Qualitative research

We are conducting up to 20 semi-structured telephone interviews with patients (10 per arm) to examine their experience of the impact of surgery and recovery period, including factors such as pain, mobility, function, independence and quality of life. Participants are sampled from across participating sites. Interviews are conducted at POD120, after the

For peer review only - http://bmjopen.bmj.com/site/about/guidelines.xhtml

quantitative data are collected, to gather information on patient experiences over the four months after their surgery. Written consent to take part in a telephone interview is included within the main trial consent form and confirmed verbally with patients when contacted for interview. Patients who lack capacity to consent are not contacted. The interview schedule is designed with advice from the patient and public involvement group, and two physiotherapists. Data will be analysed using thematic analysis (10), with NVivo 12 (11). The analysis will employ a combined deductive and inductive approach, and be underpinned by a critical realist perspective. We will triangulate the qualitative findings with quantitative findings on mobility, function and quality of life.

Patient safety and reporting

This trial follows Sponsor (Royal Devon and Exeter NHS Trust) standard operating procedures (SOPs) on safety reporting. Serious Adverse Events (SAE) within this study are defined as any untoward medical occurrence that:

- Results in death;
- Is life-threatening;
- Requires inpatient hospitalization or prolongation of existing hospitalization;
- Results in persistent or significant disability/incapacity;
- Other ‘important medical events’ may also be considered serious if they jeopardize the participant or require an intervention to prevent one of the above consequences.

All SAEs will be reported to Exeter CTU and the local site Research and Development team within 24hrs of the PI being aware of the event. Exeter CTU follows up all SAEs. An SAE occurring to a participant is reported to the research ethics committee (REC) where, in the opinion of the CI the event was ‘related’ (resulted from administration of any of the research procedures) and ‘unexpected’ in relation to those procedures, within 15 working days of the CI becoming aware of the event. Any adverse event that does not fit the definition of serious above is recorded and reported in 6 monthly safety reports to the Independent Data Monitoring Committee/Trial Steering Committee (IDMC/TSC).

Sample size

The primary outcome is the OHS measured at POD120. This gives a score between 0 and 48 (12, 13). The minimal clinically important difference for OHS when comparing two groups has been estimated to be 5 points, with a standard deviation (SD) of 9 points (14). If we conservatively use a SD of 10 (i.e. an effect size of 0.5), with 90% power, based on a t-test of two independent means at the 5% level of significance, we require 85 patients per trial arm, i.e. a total of 170. Allowing for 25% drop-out, the total recruitment target is 228 (114 in each arm). On average, each participating site is expected to recruit around 38 patients in total, or around two to three patients per month, over 18 months.

Monitoring recruitment and follow-up – internal pilot phase

For peer review only - http://bmjopen.bmj.com/site/about/guidelines.xhtml

Each site is expected to recruit two to three participants per month, but is allowed to recruit more participants if they are able to. The total target number recruited at six months is 72 participants. If the total number of participants at 6 months is 60 or more, the study will continue as planned. If the total number of participants at 6 months is less than 30, the trial will be stopped. If the number is between 30 and 60 participants, we will review procedures to see what improvements might be made, and discuss progress with the IDMC/TSC and the funder. Follow-up rates will also be reviewed at six months and if below what has been predicted (i.e. 75%) we will review procedures and see what improvements might be made. If recruitment is progressing as expected, but follow-up rates are not as expected at 10 months the IDMC/TSC will be asked to decide if the trial should continue.

Statistical analysis

The primary analyses are pre-specified and a statistical analysis plan will be drafted and agreed by the IDMC/TSC and signed off by the independent statistician on the IDMC/TSC, prior to analyses. The study will be reported in accordance with the principles of Consolidated Standards Of Reporting Trials (CONSORT) guidelines (15). There are no planned interim analyses. Primary analyses will be conducted on an intention-to-treat basis, blinded to group allocation.

The primary outcome, OHS at POD120, will be compared between study arms using linear regression, adjusting for site, cognitive impairment and pre-fracture characteristics (age, gender, place of residence, co-morbidities (ASA grouped into categories: 1 or 2, 3, 4+)). Continuous secondary outcomes (DEMMI(16), cumulative ambulatory score (CAS) and numeric pain rating scale (NPRS) at POD3; DEMMI, EQ-5D-5L, NPRS at POD 120; acute and total length of stay) will be compared between study arms using linear regression, using the same adjustment variables. Results will be presented as means and standard deviations in the two study arms and estimated mean differences with 95 percent confidence intervals and p values. As we expect length of stay to be skewed, we will check the validity of the confidence intervals for that outcome using bootstrap methods. Frequencies of death and surgical complications (by type) within 120 days follow up will be presented, in the two study arms. If there is no clear evidence of non-proportional hazards, Cox regression will be used to analyse time to death, and time to complication. Discharge destination (i.e. whether the same as pre-fracture place of residence), place of residence at 120 days (whether the same as pre-fracture place of residence) will be compared between study arms using logistic regression. For each of these outcomes, the number of events will be checked when considering adjustment factors to be included in the analyses.
Unadjusted analyses of the primary outcome and secondary outcomes will also be run. Analysis of safety outcomes (including operative complications) will be based on the per-protocol population as well as on an intention-to-treat basis.

Data Management and confidentiality

For peer review only - http://bmjopen.bmj.com/site/about/guidelines.xhtml

Data collection tools and source document identification

The data collection tool for this study is paper Case Report Forms (CRFs). Data are entered directly onto the CRFs and considered source documents. The CRF consists of standardised outcome measures (listed in table 1).

Data handling and record keeping

The recruitment sites store all original signed informed consent forms and copies of the CRF pages. Information on these documents is transcribed at site to a trial database. The CTU Trial Manager checks the trial database for data completeness and liaises with sites regarding any data queries.

Access to Data

Direct access is granted to authorised representatives from the Sponsor, host institution and the regulatory authorities to permit trial-related monitoring, audits and inspections in line with participant consent.

Data protection and patient confidentiality

The trial ID is used to identify data collected on CRFs and stored on the CTU database. Access to the CTU database is password protected and limited to those individuals necessary for quality control, audit, and analyses. The sponsor acts as the data controller for this study and will archive identifiable information for up to five years after the study has finished. Non-identifiable information will be kept in an open access archive managed by Exeter University indefinitely.

Archiving

Archiving is authorised by the Sponsor following submission of the end of trial report. The sponsor is responsible for archiving the essential documents. Exeter CTU is responsible for archiving the trial database. All essential documents will be archived for five years after completion of trial. Destruction of essential documents will require authorisation from the Sponsor.

Monitoring, audit and inspection

A Trial Monitoring Plan has been developed and agreed by the Trial Management Group (TMG), IDMC/TSC (for charter contact lead author) and CI based on the trial risk assessment. Monitoring is being conducted by the CTU Trial manager both remotely using the trial database and also with in person visits to sites. The CTU Trial Manager monitors participant enrolment, consent, eligibility, and allocation to trial groups; adherence to trial interventions and policies to protect participants, including reporting of harm and completeness, accuracy, and timeliness of data collection. Site staff are expected to assist the Trial Manager when requests for information are made or when an in person site visit is arranged.

For peer review only - http://bmjopen.bmj.com/site/about/guidelines.xhtml

For peer review only

Patient and public involvement

The premise and the primary outcome of this trial was informed by discussions with patients about key outcomes for a separate project. We continue to integrate the involvement of patients in this work, organised by patient Co-applicant AA and PPI facilitator EC. To date patients/carers with experience of hip fractures have helped develop the study design, plain English summary, patient facing documents and the qualitative interview schedule; they will also be closely involved in co-designing patient friendly dissemination materials.

ETHICS AND DISSEMINATION

Ethics

The protocol has been approved by the Yorkshire and the Humber – Bradford Leeds Research Ethics Committee (REC Reference: IRAS 258327). The trial is conducted in accordance with the study protocol, the principles of the Declaration of Helsinki, International Conference on Harmonisation of Good Clinical Practice and the Medicines for Human Use (Clinical Trials) Regulations, 2004 (17). Also in accordance with the UK Policy Framework for Health and Social Care Research (18) the Mental Capacity Act 2005 (19) and the Data Protection Act 2018 (20). The trial has been adopted by the National Institute for Health Research (NIHR) Clinical Research Network and has relevant local NHS research approvals. The trial is sponsored by Royal Devon and Exeter NHS Trust, and managed by the UKCRC-registered Exeter CTU.

Amendments

Sponsor SOPs are being followed for amendments. The decision to make an amendment is made by the TMG with sponsor approval. The sponsor decides whether an amendment is substantial or non-substantial. The Exeter CTU Trial Manager submits amendments to the REC, record approvals and communicate approved amendments to sites. Amendment history will be tracked and recorded in the Trial Master File maintained by Exeter CTU.

Dissemination and impact activities

Trial progress is reported at TMG and IDMC/TSC meetings. We will follow established practice in our institution in disseminating the results of the HEMISPAIRE trial using the widest range possible of peer reviewed scientific journals, professional publications, and national academic meetings. We will present at national and international conferences. If proven to be superior, the SPAIRE technique is likely to continue at recruiting sites and also adopted in other centres around the UK. Results will be incorporated into our clinical raining programmes and we will make recommendations to regulatory bodies such as NICE. At the end of the trial we will seek input from our PPI representatives to help disseminate a lay summary of the findings to study participants. Results will be disseminated via public involvement events, patient groups, networks and media releases.

For peer review only - http://bmjopen.bmj.com/site/about/guidelines.xhtml

For peer review only

ACKNOWLEDGMENTS AND FUNDING STATEMENT

This study is funded by the National Institute for Health Research (NIHR) Research for Patient Benefit (PB-PG-0817-20039). The views expressed are those of the author(s) and not necessarily those of the NIHR or the Department of Health and Social Care.

SB and EC are supported by the National Institute for Health Research Applied Research Collaboration South West Peninsula. The views expressed in this publication are those of the authors and not necessarily those of the National Institute for Health Research or the Department of Health and Social Care.

Author declarations of competing interests

Dr Price, Dr Ball, Dr Gordon, Dr Timperley, Dr Aylward, Dr Wickins, Dr Powell, Dr Morgan-Trimmer, and Dr Cockcroft have nothing to disclose. Dr. Rhodes reports grants from NIHR RFPB, during the conduct of the study. Dr Charity reports grants from NIHR, during the conduct of the study.

We would like to thank all those who have contributed to this study, including; the patients and careers who have been involved in the conception and planning of this research; colleagues for their advice and support. Also the PIs, surgeon collaborators, and research teams across the NHS Trust sites that have been involved in this study: Royal Devon and Exeter, Royal Cornwall Hospitals, Northern Devon Healthcare, Somerset, Yeovil Hospital and Weston Area Health.

Author contributions

The research idea was inspired by hip hemiarthroplasty patients. All co-applicants and external advisors actively contributed with the study design. SR, SB and JC developed the protocol. AP drafted the protocol ready for publication, manages the trial, and wrote amendments for ethics submission. JC leads the trial. RW is responsible for cross-site training on outcome measures and LG is lead nurse for the lead site. SB is the trial statistician. AA leads on patient and public involvement (PPI) representation. EC provides PPI expertise. RP reviewed the manuscript: he has been a RfPB-funded trial CI and has statistical skills, design and ethics committee experience. SMT provides qualitative research expertise, supported by EC. SR provides senior trial management expertise, supported by AP as trial manager. All authors commented on the protocol and the manuscript.

For peer review only

REFERENCES

1. **RCP**. Royal College of Physicians. National Hip Fracture Database annual report 2017 London2016 [cited 2020. Available from: https://www.nhfd.co.uk/2017report.
2. McGilton KS, Davis AM, Naglie G, Mahomed N, Flannery J, Jaglal S, et al. Evaluation of patient-centered rehabilitation model targeting older persons with a hip fracture, including those with cognitive impairment. BMC geriatrics. 2013;13(1):136.
3. **RCP**. Royal College of Physicians. National Hip Fracture Database annual report 2016 London2016 [cited 2020. Available from: https://www.rcplondon.ac.uk/projects/outputs/national-hip-fracture-database-annual-report-2016.
4. NICE. Hip fracture: the management of hip fracture in adults (CG124) 2011 [cited 2020. Available from: https://www.nice.org.uk/guidance/cg124.
5. Kristensen TB, Vinje T, Havelin LI, Engesæter LB, Gjertsen J-E. Posterior approach compared to direct lateral approach resulted in better patient-reported outcome after hemiarthroplasty for femoral neck fracture: 20,908 patients from the Norwegian Hip Fracture Register. Acta orthopaedica. 2017;88(1):29-34.
6. Han SK, Kim YS, Kang SH. Treatment of femoral neck fractures with bipolar hemiarthroplasty using a modified minimally invasive posterior approach in patients with neurological disorders. Orthopedics. 2012;35(5):e635-e40.
7. Vaarbakken K, Steen H, Samuelsen G, Dahl HA, Leergaard TB, Nordsletten L, et al. Lengths of the external hip rotators in mobilized cadavers indicate the quadriceps coxa as a primary abductor and extensor of the flexed hip. Clinical Biomechanics. 2014;29(7):794-802.
8. Fullam J, Theodosi PG, Charity J, Goodwin VA. A scoping review comparing two common surgical approaches to the hip for hemiarthroplasty. BMC surgery. 2019;19(1):32.
9. Parker MJ. Lateral versus posterior approach for insertion of hemiarthroplasties for hip fractures: A randomised trial of 216 patients. Injury. 2015;46(6):1023-7.
10. Braun V, Clarke V. Using thematic analysis in psychology. Qualitative research in psychology. 2006;3(2):77-101.
11. QSR International Pty Ltd. NVivo qualitative data analysis software, version 12. 2018.
12. Dawson J, Fitzpatrick R, Carr A, Murray D. Questionnaire on the perceptions of patients about total hip replacement. The Journal of bone and joint surgery British volume. 1996;78(2):185-90.
13. Murray D, Fitzpatrick R, Rogers K, Pandit H, Beard D, Carr A, et al. The use of the Oxford hip and knee scores. The Journal of bone and joint surgery British volume. 2007;89(8):1010-4.
14. Beard DJ, Harris K, Dawson J, Doll H, Murray DW, Carr AJ, et al. Meaningful changes for the Oxford hip and knee scores after joint replacement surgery. Journal of clinical epidemiology. 2015;68(1):73-9.
15. Schulz KF, Altman DG, Moher D. CONSORT 2010 statement: updated guidelines for reporting parallel group randomised trials. BMC medicine. 2010;8(1):18.
16. de Morton NA, Harding KE, Taylor NF, Harrison G. Validity of the de Morton Mobility Index (DEMMI) for measuring the mobility of patients with hip fracture during rehabilitation. Disability and rehabilitation. 2013;35(4):325-33.
17. Statutory Instrument No 1031. Medicines for Human Use (Clinical Trials) Regulations 2004. London: The Stationery Office; 2004.

For peer review only - http://bmjopen.bmj.com/site/about/guidelines.xhtml

18. NHS Health Research Authority. UK policy framework for health and social care research. London: Health Research Authority 2017.
19. Statutory Instrument. Mental Capacity Act 2005. London: The Stationery Office; 2005.
20. Statutory Instrument. Data Protection Act 2018. London: The Stationery Office; 2018.

Figure legend

Figure 1: Flow chart of trial design, with anticipated numbers of participants

For peer review only

For peer review only - http://bmjopen.bmj.com/site/about/guidelines.xhtml

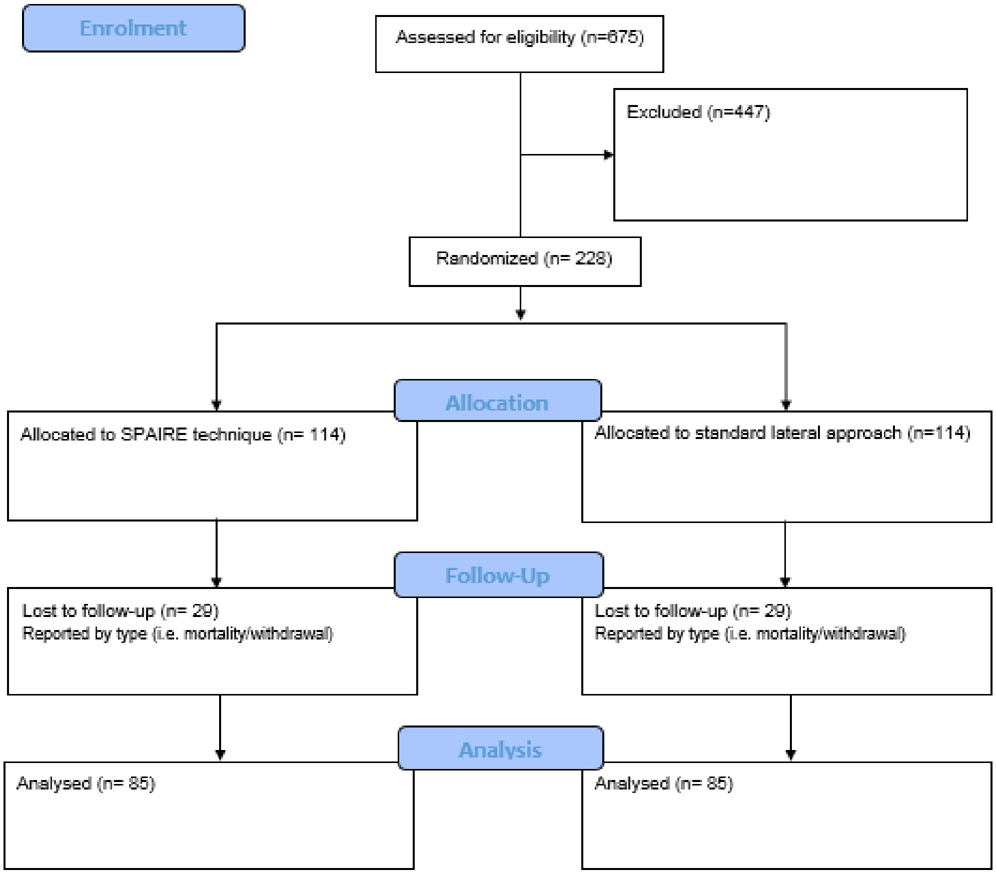

Figure 1: Flow chart of trial design, with anticipated numbers of participants

STANDARD PROTOCOL ITEMS: RECOMMENDATIONS FOR INTERVENTIONAL TRIALS

SPIRIT 2013 Checklist for the HemiSPAIRE study: Recommended items to address in a clinical trial protocol and related documents*

Section/item	Item No	Description	Addressed on page number
**Administrative information**			
Title	1	Descriptive title identifying the study design, population, interventions, and, if applicable, trial acronym	1
Trial registration	2a	Trial identifier and registry name. If not yet registered, name of intended registry	2
	2b	All items from the World Health Organization Trial Registration Data Set	Yes, clinicaltrials.gov
Protocol version	3	Date and version identifier	1
Funding	4	Sources and types of financial, material, and other support	14,15
Roles and responsibilities	5a	Names, affiliations, and roles of protocol contributors	1&14
	5b	Name and contact information for the trial sponsor	1
	5c	Role of study sponsor and funders, if any, in study design; collection, management, analysis, and interpretation of data; writing of the report; and the decision to submit the report for publication, including whether they will have ultimate authority over any of these activities	14
	5d	Composition, roles, and responsibilities of the coordinating centre, steering committee, endpoint adjudication committee, data management team, and other individuals or groups overseeing the trial, if applicable (see Item 21a for data monitoring committee)	12,13,14

Commented [PA1]: Check page numbers after final edit

For peer review only - http://bmjopen.bmj.com/site/about/guidelines.xhtml

**Introduction**			
Background and rationale	6a	Description of research question and justification for undertaking the trial, including summary of relevant studies (published and unpublished) examining benefits and harms for each intervention	4, 5
	6b	Explanation for choice of comparators	5
Objectives	7	Specific objectives or hypotheses	5, 6
Trial design	8	Description of trial design including type of trial (eg, parallel group, crossover, factorial, single group), allocation ratio, and framework (eg, superiority, equivalence, noninferiority, exploratory)	5, 6
**Methods: Participants, interventions, and outcomes**			
Study setting	9	Description of study settings (eg, community clinic, academic hospital) and list of countries where data will be collected. Reference to where list of study sites can be obtained	1, 6
Eligibility criteria	10	Inclusion and exclusion criteria for participants. If applicable, eligibility criteria for study centres and individuals who will perform the interventions (eg, surgeons, psychotherapists)	7
Interventions	11a	Interventions for each group with sufficient detail to allow replication, including how and when they will be administered	8
	11b	Criteria for discontinuing or modifying allocated interventions for a given trial participant (eg, drug dose change in response to harms, participant request, or improving/worsening disease)	10
	11c	Strategies to improve adherence to intervention protocols, and any procedures for monitoring adherence (eg, drug tablet return, laboratory tests)	10, 11
	11d	Relevant concomitant care and interventions that are permitted or prohibited during the trial	10, 11
Outcomes	12	Primary, secondary, and other outcomes, including the specific measurement variable (eg, systolic blood pressure), analysis metric (eg, change from baseline, final value, time to event), method of aggregation (eg, median, proportion), and time point for each outcome. Explanation of the clinical relevance of chosen efficacy and harm outcomes is strongly recommended	Table 1, (9)
Participant timeline	13	Time schedule of enrolment, interventions (including any run-ins and washouts), assessments, and visits for participants. A schematic diagram is highly recommended (see Figure)	Table 1 (9) & Figure 1 (6), 7, 8

For peer review only - http://bmjopen.bmj.com/site/about/guidelines.xhtml

Sample size	14	Estimated number of participants needed to achieve study objectives and how it was determined, including clinical and statistical assumptions supporting any sample size calculations	10,11
Recruitment	15	Strategies for achieving adequate participant enrolment to reach target sample size	11
**Methods: Assignment of interventions (for controlled trials)**			
Allocation:			
Sequence generation	16a	Method of generating the allocation sequence (eg, computer-generated random numbers), and list of any factors for stratification. To reduce predictability of a random sequence, details of any planned restriction (eg, blocking) should be provided in a separate document that is unavailable to those who enrol participants or assign interventions	7, 8
Allocation concealment mechanism	16b	Mechanism of implementing the allocation sequence (eg, central telephone; sequentially numbered, opaque, sealed envelopes), describing any steps to conceal the sequence until interventions are assigned	11
Implementation	16c	Who will generate the allocation sequence, who will enrol participants, and who will assign participants to interventions	7,8,11,12
Blinding (masking)	17a	Who will be blinded after assignment to interventions (eg, trial participants, care providers, outcome assessors, data analysts), and how	8
	17b	If blinded, circumstances under which unblinding is permissible, and procedure for revealing a participant's allocated intervention during the trial	8,10, 12
**Methods: Data collection, management, and analysis**			
Data collection methods	18a	Plans for assessment and collection of outcome, baseline, and other trial data, including any related processes to promote data quality (eg, duplicate measurements, training of assessors) and a description of study instruments (eg, questionnaires, laboratory tests) along with their reliability and validity, if known. Reference to where data collection forms can be found, if not in the protocol	Table 1 (9) 11, 12, 13
	18b	Plans to promote participant retention and complete follow-up, including list of any outcome data to be collected for participants who discontinue or deviate from intervention protocols	Table 1 (9), 12, 13

Data management	19	Plans for data entry, coding, security, and storage, including any related processes to promote data quality (eg, double data entry; range checks for data values). Reference to where details of data management procedures can be found, if not in the protocol	12,13
Statistical methods	20a	Statistical methods for analysing primary and secondary outcomes. Reference to where other details of the statistical analysis plan can be found, if not in the protocol	11, 12
	20b	Methods for any additional analyses (eg, subgroup and adjusted analyses)	11, 12
	20c	Definition of analysis population relating to protocol non-adherence (eg, as randomised analysis), and any statistical methods to handle missing data (eg, multiple imputation)	11, 12
**Methods: Monitoring**			
Data monitoring	21a	Composition of data monitoring committee (DMC); summary of its role and reporting structure; statement of whether it is independent from the sponsor and competing interests; and reference to where further details about its charter can be found, if not in the protocol. Alternatively, an explanation of why a DMC is not needed	10, 11, 12
	21b	Description of any interim analyses and stopping guidelines, including who will have access to these interim results and make the final decision to terminate the trial	11
Harms	22	Plans for collecting, assessing, reporting, and managing solicited and spontaneously reported adverse events and other unintended effects of trial interventions or trial conduct	10
Auditing	23	Frequency and procedures for auditing trial conduct, if any, and whether the process will be independent from investigators and the sponsor	11
**Ethics and dissemination**			
Research ethics approval	24	Plans for seeking research ethics committee/institutional review board (REC/IRB) approval	1, 13
Protocol amendments	25	Plans for communicating important protocol modifications (eg, changes to eligibility criteria, outcomes, analyses) to relevant parties (eg, investigators, REC/IRBs, trial participants, trial registries, journals, regulators)	12

For peer review only - http://bmjopen.bmj.com/site/about/guidelines.xhtml

Consent or assent	26a	Who will obtain informed consent or assent from potential trial participants or authorised surrogates, and how (see Item 32)	7
	26b	Additional consent provisions for collection and use of participant data and biological specimens in ancillary studies, if applicable	N/A
Confidentiality	27	How personal information about potential and enrolled participants will be collected, shared, and maintained in order to protect confidentiality before, during, and after the trial	12, 13
Declaration of interests	28	Financial and other competing interests for principal investigators for the overall trial and each study site	14
Access to data	29	Statement of who will have access to the final trial dataset, and disclosure of contractual agreements that limit such access for investigators	14
Ancillary and post-trial care	30	Provisions, if any, for ancillary and post-trial care, and for compensation to those who suffer harm from trial participation	N/A
Dissemination policy	31a	Plans for investigators and sponsor to communicate trial results to participants, healthcare professionals, the public, and other relevant groups (eg, via publication, reporting in results databases, or other data sharing arrangements), including any publication restrictions	13
	31b	Authorship eligibility guidelines and any intended use of professional writers	14
	31c	Plans, if any, for granting public access to the full protocol, participant-level dataset, and statistical code	
**Appendices**			
Informed consent materials	32	Model consent form and other related documentation given to participants and authorised surrogates	Available on request
Biological specimens	33	Plans for collection, laboratory evaluation, and storage of biological specimens for genetic or molecular analysis in the current trial and for future use in ancillary studies, if applicable	n/a

*It is strongly recommended that this checklist be read in conjunction with the SPIRIT 2013 Explanation & Elaboration for important clarification on the items. Amendments to the protocol should be tracked and dated. The SPIRIT checklist is copyrighted by the SPIRIT Group under the Creative Commons "Attribution-NonCommercial-NoDerivs 3.0 Unported" license.

For peer review only - http://bmjopen.bmj.com/site/about/guidelines.xhtml

a) WHO Trial Registration Data Set (Version 1.2.1)

The minimum amount of trial information that must appear in a register in order for a given trial to be considered fully registered. There are currently 20 items in the WHO Trial Registration Data Set. It is sometimes referred to as the TRDS.

- **Primary Registry and Trial Identifying Number**
  Name of Primary Registry, and the unique ID number assigned by the Primary Registry to this trial.

- **Date of Registration in Primary Registry**
  Date when trial was officially registered in the Primary Registry.

- **Secondary Identifying Numbers**
  Other identifiers besides the Trial Identifying Number allocated by the Primary Registry, if any. These include:
  - The Universal Trial Number (UTN)
  - Identifiers assigned by the sponsor (record Sponsor name and Sponsor-issued trial number (e.g. protocol number))
  - Other trial registration numbers issued by other Registries (both Primary and Partner Registries in the WHO Registry Network, and other registries)
  - Identifiers issued by funding bodies, collaborative research groups, regulatory authorities, ethics committees / institutional review boards, etc.

  All secondary identifiers will have 2 elements: an identifier for the issuing authority (e.g. NCT, ISRCTN, ACTRN) plus a number.

  There is no limit to the number of secondary identifiers that can be provided.

- **Source(s) of Monetary or Material Support**
  Major source(s) of monetary or material support for the trial (e.g. funding agency, foundation, company, institution).

- **Primary Sponsor**
  The individual, organization, group or other legal entity which takes responsibility for initiating, managing and/or financing a study. The Primary Sponsor is responsible for ensuring that the trial is properly registered. The Primary Sponsor may or may not be the main funder.

- **Secondary Sponsor(s)**
  Additional individuals, organizations or other legal persons, if any, that have agreed with the primary sponsor to take on responsibilities of sponsorship.

  A secondary sponsor may have agreed to:
  - take on all the responsibilities of sponsorship jointly with the primary sponsor; or
  - form a group with the Primary Sponsor in which the responsibilities of sponsorship are allocated among the members of the group; or

For peer review only

- act as the Primary Sponsor's legal representative in relation to some or all of the trial sites.

- **Contact for Public Queries**
  Email address, telephone number and postal address of the contact who will respond to general queries, including information about current recruitment status.

- **Contact for Scientific Queries**
  There must be clearly assigned responsibility for scientific leadership to a named Principal Investigator. The PI may delegate responsibility for dealing with scientific enquiries to a scientific contact for the trial. This scientific contact will be listed in addition to the PI.

  The contact for scientific queries must therefore include:
  - Name and title, email address, telephone number, postal address and affiliation of the Principal Investigator, and;
  - Email address, telephone number, postal address and affiliation of the contact for scientific queries about the trial (if applicable). The details for the scientific contact may be generic (that is, there does not need to be a named individual): e.g. a generic email address for research team members qualified to answer scientific queries.

- **Public Title**
  Title intended for the lay public in easily understood language.

- **Scientific Title**
  Scientific title of the study as it appears in the protocol submitted for funding and ethical review. Include trial acronym if available.

- **Countries of Recruitment**
  The countries from which participants will be, are intended to be, or have been recruited at the time of registration.

- **Health Condition(s) or Problem(s) Studied**
  Primary health condition(s) or problem(s) studied (e.g., depression, breast cancer, medication error).

  If the study is conducted in healthy human volunteers belonging to the target population of the intervention (e.g. preventive or screening interventions), enter the particular health condition(s) or problem(s) being prevented.

- **Intervention(s)**
  For each arm of the trial record a brief intervention name plus an intervention description.

  Intervention Name: For drugs use generic name; for other types of interventions provide a brief descriptive name.
  - For investigational new drugs that do not yet have a generic name, a chemical name, company code or serial number may be used on a temporary basis. As soon as the generic name has been established, update the associated registered records accordingly.

For peer review only - http://bmjopen.bmj.com/site/about/guidelines.xhtml

  - For non-drug intervention types, provide an intervention name with sufficient detail so that it can be distinguished from other similar interventions.

Intervention Description: Must be sufficiently detailed for it to be possible to distinguish between the arms of a study (e.g. comparison of different dosages of drug) and/or among similar interventions (e.g. comparison of multiple implantable cardiac defibrillators). For example, interventions involving drugs may include dosage form, dosage, frequency and duration.

If the intervention is one or more drugs then use the International Non-Proprietary Name for each drug if possible (not brand/trade names). For an unregistered drug, the generic name, chemical name, or company serial number is acceptable.

If the intervention consists of several separate treatments, list them all in one line separated by commas (e.g. "low-fat diet, exercise").

For controlled trials, the identity of the control arm should be clear. The control intervention(s) is/are the interventions against which the study intervention is evaluated (e.g. placebo, no treatment, active control). If an active control is used, be sure to enter in the name(s) of that intervention, or enter "placebo" or "no treatment" as applicable. For each intervention, describe other intervention details as applicable (dose, duration, mode of administration, etc).

- **Key Inclusion and Exclusion Criteria**
  Inclusion and exclusion criteria for participant selection, including age and sex. Other selection criteria may relate to clinical diagnosis and co-morbid conditions; exclusion criteria are often used to ensure patient safety.

  If the study is conducted in healthy human volunteers not belonging to the target population (e.g. a preliminary safety study), enter "healthy human volunteer".

- **Study Type**
  Study type consists of:
  - Type of study (interventional or observational)
  - Study design including:
    - Method of allocation (randomized/non-randomized)
    - Masking (is masking used and, if so, who is masked)
    - Assignment (single arm, parallel, crossover or factorial)
    - Purpose
  - Phase (if applicable)

For peer review only - http://bmjopen.bmj.com/site/about/guidelines.xhtml

For randomized trials: the allocation concealment mechanism and sequence generation will be documented.

- **Date of First Enrollment**
  Anticipated or actual date of enrolment of the first participant.

- **Target Sample Size**
  Number of participants that this trial plans to enrol in total.

- **Recruitment Status**
  Recruitment status of this trial:
  - Pending: participants are not yet being recruited or enrolled at any site
  - Recruiting: participants are currently being recruited and enrolled
  - Suspended: there is a temporary halt in recruitment and enrolment
  - Complete: participants are no longer being recruited or enrolled
  - Other

- **Primary Outcome(s)**
  Outcomes are events, variables, or experiences that are measured because it is believed that they may be influenced by the intervention.

  The Primary Outcome should be the outcome used in sample size calculations, or the main outcome(s) used to determine the effects of the intervention(s). Most trials should have only one primary outcome.

  For each primary outcome provide:
  - The name of the outcome (do not use abbreviations)
  - The metric or method of measurement used (be as specific as possible)
  - The timepoint(s) of primary interest

  Example:
  Outcome Name: Depression
  Metric/method of measurement: Beck Depression Score
  Timepoint: 18 weeks following end of treatment

- **Key Secondary Outcomes**
  Secondary outcomes are outcomes which are of secondary interest or that are measured at timepoints of secondary interest. A secondary outcome may

For peer review only

involve the same event, variable, or experience as the primary outcome, but measured at timepoints other than those of primary interest.

As for primary outcomes, for each secondary outcome provide:

- The name of the outcome (do not use abbreviations)
- The metric or method of measurement used (be as specific as possible)
- The timepoint(s) of interest

For peer review only

For peer review only - http://bmjopen.bmj.com/site/about/guidelines.xhtml

The effects of a modified muscle sparing posterior technique in hip hemiarthroplasty for displaced intracapsular fractures on post-operative function compared to a standard lateral approach (HemiSPAIRE); protocol for a randomised controlled trial.

Journal:	*BMJ Open*
Manuscript ID	bmjopen-2020-045652.R1
Article Type:	Protocol
Date Submitted by the Author:	30-Mar-2021
Complete List of Authors:	Price, Anna; University of Exeter Medical School, Clinical Trials Unit<br>Ball, Susan; University of Exeter Medical School, NIHR CLAHRC South West Peninsula (PenCLAHRC)<br>Rhodes, Shelley; University of Exeter Medical School, Clinical Trials Unit<br>Wickins, Robert; Royal Devon and Exeter NHS Foundation Trust, Physiotherapy<br>Gordon, Elizabeth; Royal Devon and Exeter NHS Foundation Trust, Research<br>Aylward, Alex; University of Exeter Medical School, Public and Patient Involvement Group<br>Cockcroft, Emma; University of Exeter Medical School<br>Morgan-Trimmer, Sarah; University of Exeter Medical School, Psychology Applied to Health (PAtH) Group, Institute of Health Research<br>Powell, Roy; Royal Devon and Exeter NHS Foundation Trust, Research Design Service<br>Timperley, John; Royal Devon and Exeter NHS Foundation Trust, Exeter, Hip Unit, Princess Elizabeth Orthopaedic Centre<br>Charity, John; Royal Devon and Exeter NHS Foundation Trust, Exeter, Hip Unit, Princess Elizabeth Orthopaedic Centre
**Primary Subject Heading**:	Surgery
Secondary Subject Heading:	Surgery, Patient-centred medicine, Health services research, Evidence based practice
Keywords:	Hip < ORTHOPAEDIC & TRAUMA SURGERY, SURGERY, ORTHOPAEDIC & TRAUMA SURGERY, Clinical trials < THERAPEUTICS

SCHOLARONE™
Manuscripts

I, the Submitting Author has the right to grant and does grant on behalf of all authors of the Work (as defined in the below author licence), an exclusive licence and/or a non-exclusive licence for contributions from authors who are: i) UK Crown employees; ii) where BMJ has agreed a CC-BY licence shall apply, and/or iii) in accordance with the terms applicable for US Federal Government officers or employees acting as part of their official duties; on a worldwide, perpetual, irrevocable, royalty-free basis to BMJ Publishing Group Ltd ("BMJ") its licensees and where the relevant Journal is co-owned by BMJ to the co-owners of the Journal, to publish the Work in this journal and any other BMJ products and to exploit all rights, as set out in our licence.

The Submitting Author accepts and understands that any supply made under these terms is made by BMJ to the Submitting Author unless you are acting as an employee on behalf of your employer or a postgraduate student of an affiliated institution which is paying any applicable article publishing charge ("APC") for Open Access articles. Where the Submitting Author wishes to make the Work available on an Open Access basis (and intends to pay the relevant APC), the terms of reuse of such Open Access shall be governed by a Creative Commons licence – details of these licences and which Creative Commons licence will apply to this Work are set out in our licence referred to above.

Other than as permitted in any relevant BMJ Author's Self Archiving Policies, I confirm this Work has not been accepted for publication elsewhere, is not being considered for publication elsewhere and does not duplicate material already published. I confirm all authors consent to publication of this Work and authorise the granting of this licence.

For peer review only

TITLE: The effects of a modified muscle sparing posterior technique in hip hemiarthroplasty for displaced intracapsular fractures on post-operative function compared to a standard lateral approach (HemiSPAIRE); protocol for a randomised controlled trial.

Authors

Anna Price[1], Susan Ball[2], Shelley Rhodes[1], Robert Wickins[3], Elizabeth Gordon[3], Alex Aylward[2], Emma Cockcroft[2], Sarah Morgan-Trimmer[1], Roy Powell[1, 3], John Timperley[1, 3], John Charity[3].

[1]University of Exeter Medical School, Exeter, EX1 2LU, UK.
[2]NIHR Applied Research Collaboration South West Peninsula, University of Exeter Medical School, Exeter, EX1 2LU, UK.
[3]Royal Devon & Exeter Hospital, Exeter, EX2 5DW, UK.

Corresponding author
Dr Anna Price,
Clinical Trials Unit,
University of Exeter Medical School,
College House
St Luke's Campus,
Exeter,
EX1 2LU,
Email: a.price@exeter.ac.uk
01392 726026

Trial sponsor
Dr Alison Kerridge,
Assistant R&D Manager,
NIHR Clinical Research Network: South West Peninsula
Room F6, Bowmoor House,
Royal Devon & Exeter NHS Foundation Trust, Barrack Road,
Exeter, EX2 5DW
Email: alison.kerridge @nhs.net
01392 403055

Key words: Displaced intracapsular hip fracture, Hip hemiarthroplasty, Surgical approach, Muscle sparing, Function, Clinical Trials.

Protocol Version: 2 Date: 13/05/2020

Word count: 3980

For peer review only - http://bmjopen.bmj.com/site/about/guidelines.xhtml

ABSTRACT

Introduction: Currently National Institute for Health and Care Excellence clinical guidelines in the United Kingdom suggest that surgeons performing partial hip replacements (hemiarthroplasty) should consider using the lateral approach. Alternatively, a newer, modified posterior approach using a muscle sparing technique named 'Save Piriformis and Internus, Repairing Externus' (SPAIRE) can be used leaving the major muscles intact. This randomised controlled trial (RCT) aims to compare the SPAIRE approach to the standard lateral approach, to determine if it allows patients to mobilise better and experience improved function after surgery.

Methods and analysis: HemiSPAIRE is a two-arm, assessor-blinded, definitive pragmatic RCT with nested pilot and qualitative studies. Two-hundred and twenty eight participants with displaced intracapsular fractures requiring hip hemiarthroplasty will be individually randomised 1:1 to either the SPAIRE, or control (standard lateral approach) surgical procedure. Outcomes will be assessed at post-operative day three (POD3), and one-hundred and twenty (POD120). The primary outcome measure will be level of function and mobility using the Oxford Hip Score (OHS) at POD120. Secondary outcomes include: De Morton Mobility Index (DEMMI), Cumulated Ambulatory Score (CAS) and Numeric Pain Rating Scale (NPRS) at POD3; DEMMI, NPRS and EQ-5D-5L at POD120, complications, acute and total length of hospital stay, and mortality. Primary analysis will be on an intention-to-treat basis. Participant experiences of the impact of surgery and recovery period will be examined via up to twenty semi-structured telephone interviews.

Ethics and dissemination: The protocol has been approved by Yorkshire and the Humber – Bradford Leeds Research Ethics Committee. Recruitment commenced in November 2019. Findings will be disseminated via research articles in peer-reviewed journals, presentations at conferences, public involvement events, patient groups, and media releases. A summary of the trial findings will be shared with participants at the end of the study.

Registration: ClinicalTrials.gov: trial number NCT04095611.

For peer review only - http://bmjopen.bmj.com/site/about/guidelines.xhtml

ARTICLE SUMMARY

Strengths and limitations of this study

- Comprehensive Patient and Public Involvement: outcome measures were inspired by patients who said that mobility and speed to regaining independence are the most important outcomes after partial hip replacement surgery
- Addresses the current evidence gap on the impact on patient mobilisation and function after surgery using different techniques (SPAIRE vs. lateral)
- A pragmatic multicentre study across six NHS Trusts in the South West of England with broad inclusion criteria to recruit a population that will mirror as close as possible this frail group of patients as reduced cognition is not an exclusion criterion
- Due to the frail characteristics of the target population a relatively high dropout rate is anticipated, however this has been accounted for in the sample size calculation
- Due to Covid19, the primary outcome measure was changed to a self-report measure, meaning some outcomes were not collected in the manner originally planned

For peer review only

INTRODUCTION

Hip fractures are common in the elderly. Over 20,000 cases of hip hemiarthroplasty (replacement of the fractured femoral head) are performed annually in England, Wales and Northern Ireland (1). Hip fracture patients endure debilitating loss of function, and recovery is often complex and challenging (2). The average total length of stay for hip fracture admissions is over 21 days, representing over 4000 National Health Service (NHS) hospital beds occupied by hip fracture patients at any one time (3).

The National Institute for Health and Care Excellence (NICE) recommends replacement arthroplasty (total hip replacement or hemiarthroplasty) to patients with a displaced intracapsular hip fracture. For patients who are not eligible for total hip replacement, hemiarthroplasty should be offered.(4). As a commonly performed procedure, it is important to consider innovative hemiarthroplasty techniques that may allow better and safer rehabilitation in this frail group of patients.

When planning a hip hemiarthroplasty, surgeons have a choice of surgical approaches. NICE guidelines (4) currently recommend using a lateral rather than a conventional posterior approach. This advice is based on evidence described as being of “very poor quality” (4). For adequate exposure, the lateral approach requires division and subsequent repair of 50% or more of the tendon attachments of the gluteus medius and minimus muscles on to the greater trochanter. These muscles are essential for normal gait. Literature quoted suggests a reduced dislocation rate for the lateral approach; however this surgery has the disadvantage that the relatively extensive division of tendon attachments required may result in reduced levels of function postoperatively. Recent evidence from a cohort of over 20,000 patients from the Norwegian Hip Fracture Register (5) reported better patient-related outcome measures (pain, patient satisfaction and health-related quality of life) with a standard posterior approach compared to the lateral approach. However, the study also confirmed the higher dislocation rates in the conventional posterior approach group. Some studies report dislocation in up to 10% of patients undergoing this hip procedure through a standard posterior approach and such complications can lead to potentially catastrophic consequences (6).

To address the issue of dislocation in hip arthroplasty, modified surgical procedures have been attempted using muscle sparing techniques. In 2012, Han et al. (6) described a modified posterior approach for use in patients with neurological disorders requiring hip hemiarthroplasty, where the piriformis, gemellus superior, obturator internus and part of quadratus femoris muscles were left intact, which combined with a standard capsule repair led to a reduced incidence of dislocation (6).

For peer review only - http://bmjopen.bmj.com/site/about/guidelines.xhtml

In 2016, the Hip Unit at the Royal Devon and Exeter NHS Foundation Trust developed a modified technique using a posterior approach for hip hemiarthroplasties applicable to all patients. This approach involves division of only the obturator externus tendon and part of quadratus femoris muscle from their femoral insertions. These are repaired, along with the posterior capsule, at the end of the surgery, with strong non-absorbable suture (as opposed to a standard repair) through an enhanced trans-osseous technique onto the posterior aspect of the greater trochanter. This is a modification of the more extensive posterior repair technique developed by Carlton Savory, MD at The Hughston Clinic, Georgia, USA. The tendon insertions of piriformis, gemellus superior, obturator internus and gemellus inferior muscles are spared, and the extensive abductor muscle insertions of gluteus medius and gluteus minimus onto the greater trochanter are left undisturbed, minimising the potential negative impact of dividing muscles during surgery on post-operative recovery and mobility. This technique is named “SPAIRE” as it allows the surgeon to “Save Piriformis and Internus, Repairing Externus”. These muscles have been shown to act as the main extensor and abductor of the flexed hip which is of significant importance for movements such as rising from a chair or climbing stairs (7). This contrasts to the standard lateral approach, where a significant proportion of the gluteal muscle insertions is divided, potentially impacting hip function. The combination of this muscle sparing approach with an enhanced capsule repair aims to provide sufficient stability to enable patients to mobilise full-weight bearing, without any of the specific restrictions currently included in routine post-operative posterior approach protocols. The preservation of these muscle insertions may replicate or even surpass the improved patient-related outcome measures observed in the study by Kristensen et al. (5) and might achieve other benefits relating to more complete rehabilitation, reduced hospital stay, and diminished requirement for social service support on discharge, with consequent savings from the health and social care budget.

Review of existing evidence

A scoping review identified thirteen studies that compared the traditional posterior approach with the lateral approach, including two randomised controlled trials (RCTs) (8). These evidence a trend for increased incidence of dislocation using the traditional posterior approach versus other approaches, with the exception of one paper describing good results with a modified version of the posterior approach (9). However, this approach was only attempted in a small subset of the population prone to these fractures. Evidence with regard to other outcomes is inconsistent and of limited quality, with few RCTs to inform guidelines(8).

Aims

HemiSPAIRE is a RCT which, with its focus on post-operative function and mobility, aims to provide high quality evidence on the relative benefits of the SPAIRE surgical technique when compared with standard lateral technique. The findings may help to inform and update current guidelines (4). In doing so it aims to contribute to improved function, mobility, and

For peer review only - http://bmjopen.bmj.com/site/about/guidelines.xhtml

quality of life outcomes for hip fracture patients, many of whom are elderly and frail, and reduce length of hospital stays.

Primary objective

To test whether the SPAIRE technique improves post-operative function and mobility at 120 days following surgery in adults with a displaced intracapsular hip fracture requiring hemiarthroplasty, compared to the standard lateral approach through conducting a definitive two-arm RCT.

Secondary objectives

Test whether the SPAIRE technique results in improved early function, mobility, pain and quality of life at 120 days, with reduced length of hospital stay, complication rates, and mortality compared to the standard lateral approach, through collecting secondary outcome measures in the trial.

Investigate how patients experience the recovery period and mechanisms of recovery after surgery, by conducting a qualitative study with a sub-sample of patients in each trial arm.

Engage the contribution of a PPI group to ensure the conduct and outputs of the study are relevant and useful to patients.

METHODS AND ANALYSIS

Trial design

This is a definitive pragmatic, multi-centre RCT in patients attending hospital with a displaced intracapsular fracture requiring hip hemiarthroplasty. Patients will be randomised to have their operation performed either by posterior approach (SPAIRE technique) or the standard lateral approach. Patients, ward staff and all research staff involved in post-operative evaluations will remain blinded to allocation. Outcomes will be recorded on the third post-operative day (POD3) and 120 days after surgery (POD120). These include measures of function, mobility, quality of life, pain, surgical complications, hospital length of stay, mortality, discharge destination, and place of residence. Figure 1 shows a flow chart of the trial design.

Trial setting

Six acute hospitals in the South West of England are recruitment centres, with a minimum of two surgeons per site. Training has been provided by the Chief Investigator (CI) and co-applicant JT, using lectures, mentorship, observations, videos, and one-to-one sessions as necessary. All participating surgeons have carried out a minimum of five cases using the posterior approach (SPAIRE technique) prior to participating in the study, and are prepared to undertake both procedures. Each participating surgeon uses both techniques. If a

For peer review only - http://bmjopen.bmj.com/site/about/guidelines.xhtml

surgeon is not in equipoise then he/she will not participate. Trainees who complete training under direct supervision of the principal investigator at each site are eligible to operate under supervision and are included on the list of surgeon collaborators.

Participant eligibility criteria

All patients requiring hemiarthroplasty for a displaced intracapsular hip fracture are considered for inclusion. Inclusion criteria: patients aged 60 years or older presenting with an intracapsular hip fracture requiring hip hemiarthroplasty (4) who are resident in the South West of England. Exclusion criteria: patients who were immobile (unable to walk) before hip fracture, patients not expected to live until POD120 due to chronic illness and receiving surgery for palliative care, and use of femoral stems not of a proven stem design, in line with recommendations set by NICE clinical guideline on hip fracture management (4). A sizeable proportion of this population suffer dementia and/or temporary delirium, and are not excluded; cognitive ability is not part of the eligibility criteria.

Trial procedures

Recruitment and participant identification

When a patient attends hospital with a confirmed intracapsular hip fracture, they are considered for inclusion. If potentially eligible the study is discussed with the patient and/or their carer(s). Surgery usually takes place 12 hours or more after admission or on the day after, providing patients with time to consider their participation. Potential participants are identified by orthopaedic surgeons involved in the study (co-applicants and collaborators) who admit patients under their care.

Consent

Identified patients interested in participating in the study are invited to read the participant information sheet and, if interested, to provide informed consent. Researchers will be present to encourage potential participants to ask questions and to ensure participants fully understand the purpose of the research, risks associated with the intervention, obligations of participation and their right to withdraw at any time. The main consent forms must be fully completed and signed before patients are accepted on to the study (see supplementary material provided). The qualitative consent form, if applicable, must be fully completed and signed between baseline and POD3. If a patient lacks capacity and is unable to consent, potential participation is discussed and consent later sought with a person whose relationship to the patient makes them suitable to act as his/her legal representative e.g. personal or professional consultee. If possible the study is discussed or communicated to the participant in a way appropriate to their understanding. Participants who lack the mental capacity to consent and do not seem in agreement with any part of the study, even if agreement has been given by another, are not included. The participant's general practitioner is notified by letter that his/her patient is participating in the study.

For peer review only - http://bmjopen.bmj.com/site/about/guidelines.xhtml

The randomisation scheme

Randomisation is undertaken as late as practically possible. There is no special preparation required in theatre, and no difference in equipment required for either surgical technique. Participants will be individually randomised to receive the SPAIRE or lateral procedure in a 1:1 ratio. Concealed allocation is determined by the UKCRC registered Exeter Clinical Trials Unit (CTU) using a validated password-protected web-based system. Allocation is based on random permuted blocks of varying size, and stratified by study site and by cognition level (impaired vs non-impaired). The surgeon is informed of allocation by the CTU via email through nhs.net mail. The CI and Principal Investigator (PI) at site are copied in.

Blinding

Patients, and research staff performing outcome assessments are blinded to treatment allocation. Surgeons and operative team are unblinded. An unblinded coordinator transcribes the surgical data to the database. There is no difference between the SPAIRE and lateral approach techniques in the following: surgical time taken, application of surgical dressing or post-operative care. For medicolegal reasons, the surgical approach used in the procedure is specified in the operation notes. A cover sheet is attached in front of the printed operation note stating that the patient is a participant in the trial and reminding the research team to avoid inadvertent unblinding to treatment allocation during post-operative assessments.

Trial treatments

Trial interventions

The two groups differ only in the surgical approach to the hemiarthroplasty. All preparation for surgery, patient positioning, skin incision, other aspects of surgery, surgical dressing and post-operative care, are the same according to current practice.

SPAIRE technique through the posterior approach to the hip

If randomised to this arm of the trial, the SPAIRE technique is performed. This involves a modified muscle-sparing posterior approach where insertions of piriformis, superior gemellus, obturator internus and inferior gemellus are spared with division of only obturator externus and part of quadratus femoris. The single divided tendon and posterior capsule are subsequently repaired with a transosseous repair to their initial position prior to closure. The insertions of the abductor muscles are left intact throughout the procedure.

LATERAL approach to the hip

If randomised to this arm of the trial, the patient is prepared and the operation performed in accordance with criteria set by the study, so as to minimise issues of standardisation with this approach. This means that the gluteus medius and minimus insertions onto the greater trochanter are partially divided anteriorly, leaving the posterior part of their insertions

For peer review only - http://bmjopen.bmj.com/site/about/guidelines.xhtml

intact. The anterior capsule is divided and subsequently repaired prior to closure, followed by repair of the detached portion of the gluteal muscles.

Baseline data collection and trial outcome assessments

Pre-fracture (baseline) participant characteristics, collected on the day of surgery, include: physical status at time of operation, measured using the American Society of Anaesthesiologists' (ASA) Physical Status Classification and level of cognition, determined by the surgical team, using the Abbreviated Mental Test (AMT). Pre-fracture mobility and quality of life measures, collected retrospectively at 3 days after surgery, include: Oxford Hip Score and EuroQol EQ-5D-5L.

For details of trial outcome assessments see table 1.

<u>Table 1: trial outcome assessments, with time-points</u>

**Objective**	**Outcome Assessment**	**Tests conducted at:**
**Primary**		
Assess postoperative function and mobility.	The Oxford Hip Score (OHS).	120 days after surgery.
**Secondary**		
Assess postoperative function and early mobility.		
	The De Morton Mobility Index (DEMMI) test.	3 and 120 days after surgery.
	The Cumulated Ambulation Score (CAS).	3 days after surgery.
Assess pain.	Numeric pain rating scale (NPRS).	3 and 120 days after surgery.
Measure quality of life.	EuroQol EQ-5D-5L.	120 days after surgery.
Assess analgesia medication use.	Analgesia medication use recorded from patient notes.	3 and 120 days after surgery.
Measure hospital stay.	Acute and total length of hospital stay.	3 and 120 days after surgery.
Assess any negative consequences of care.	Adverse Events (AE) and Serious Adverse Events (SAE), defined as any negative consequence of care	Continually throughout study participation, on average 120 days.

For peer review only - http://bmjopen.bmj.com/site/about/guidelines.xhtml

	resulting in unintended injury or illness.	
Assess complication rates.	Specific hip-related complications: dislocation, nerve injury, periprosthetic fracture, infection within 120 days of operation and need for re-operation (with reasons).	Within 120 days after surgery, recording the date of complication.
Record discharge destination	The percentage of participants who are discharged to their pre-fracture place of residence.	At time of discharge from hospital admission.
Return to place of residence	Place of residence. The percentage of participants returning to their pre-fracture place of residence.	At 120 days after surgery.
Estimate survival.	Mortality within 120 days of the operation.	Within 120 days after surgery, recording the date of death.

Qualitative research

We are conducting up to 20 semi-structured telephone interviews with patients (10 per arm) to examine their experience of the impact of surgery and recovery period, including factors such as pain, mobility, function, independence and quality of life. Participants are sampled from across participating sites. Interviews are conducted at POD120, after the quantitative data are collected, to gather information on patient experiences over the four months after their surgery. Written consent to take part in a telephone interview is included within the main trial consent form and confirmed verbally with patients when contacted for interview. Patients who lack capacity to consent are not contacted. The interview schedule is designed with advice from the patient and public involvement group, and two physiotherapists. Data will be analysed using thematic analysis (10), with NVivo 12 (11). The analysis will employ a combined deductive and inductive approach, and be underpinned by a critical realist perspective. We will triangulate the qualitative findings with quantitative findings on mobility, function and quality of life.

Patient safety and reporting

This trial follows Sponsor (Royal Devon and Exeter NHS Trust) standard operating procedures (SOPs) on safety reporting. Serious Adverse Events (SAE) within this study are defined as any untoward medical occurrence that:

- Results in death;
- Is life-threatening;
- Requires inpatient hospitalization or prolongation of existing hospitalization;

For peer review only - http://bmjopen.bmj.com/site/about/guidelines.xhtml

- Results in persistent or significant disability/incapacity;
- Other 'important medical events' may also be considered serious if they jeopardize the participant or require an intervention to prevent one of the above consequences.

All SAEs will be reported to Exeter CTU and the local site Research and Development team within 24hrs of the PI being aware of the event. Exeter CTU follows up all SAEs. An SAE occurring to a participant is reported to the research ethics committee (REC) where, in the opinion of the CI the event was 'related' (resulted from administration of any of the research procedures) and 'unexpected' in relation to those procedures, within 15 working days of the CI becoming aware of the event. Any adverse event that does not fit the definition of serious above is recorded and reported in 6 monthly safety reports to the Independent Data Monitoring Committee/Trial Steering Committee (IDMC/TSC).

Sample size

The primary outcome is the OHS measured at POD120. This gives a score between 0 and 48 (12, 13). The minimal clinically important difference for OHS when comparing two groups has been estimated to be 5 points, with a standard deviation (SD) of 9 points (14). If we conservatively use a SD of 10 (i.e. an effect size of 0.5), with 90% power, based on a t-test of two independent means at the 5% level of significance, we require 85 patients per trial arm, i.e. a total of 170. Allowing for 25% drop-out, the total recruitment target is 228 (114 in each arm). On average, each participating site is expected to recruit around 38 patients in total, or around two to three patients per month, over 18 months.

Monitoring recruitment and follow-up – internal pilot phase

Each site is expected to recruit two to three participants per month, but is allowed to recruit more participants if they are able to. The total target number recruited at six months is 72 participants. If the total number of participants at 6 months is 60 or more, the study will continue as planned. If the total number of participants at 6 months is less than 30, the trial will be stopped. If the number is between 30 and 60 participants, we will review procedures to see what improvements might be made, and discuss progress with the IDMC/TSC and the funder. Follow-up rates will also be reviewed at six months and if below what has been predicted (i.e. 75%) we will review procedures and see what improvements might be made. If recruitment is progressing as expected, but follow-up rates are not as expected at 10 months the IDMC/TSC will be asked to decide if the trial should continue.

Statistical analysis

The primary analyses are pre-specified and a statistical analysis plan will be drafted and agreed by the IDMC/TSC and signed off by the independent statistician on the IDMC/TSC, prior to analyses. The study will be reported in accordance with the principles of Consolidated Standards Of Reporting Trials (CONSORT) guidelines (15). There are no

For peer review only - http://bmjopen.bmj.com/site/about/guidelines.xhtml

planned interim analyses. Primary analyses will be conducted on an intention-to-treat basis, blinded to group allocation.

The primary outcome, OHS at POD120, will be compared between study arms using linear regression, adjusting for site, cognitive impairment and pre-fracture characteristics (age, gender, place of residence, co-morbidities (ASA grouped into categories: 1 or 2, 3, 4+)). Continuous secondary outcomes (DEMMI(16), cumulative ambulatory score (CAS) and numeric pain rating scale (NPRS) at POD3; DEMMI, EQ-5D-5L, NPRS at POD 120; acute and total length of stay) will be compared between study arms using linear regression, using the same adjustment variables. Results will be presented as means and standard deviations in the two study arms and estimated mean differences with 95 percent confidence intervals and p values. As we expect length of stay to be skewed, we will check the validity of the confidence intervals for that outcome using bootstrap methods. Frequencies of death and surgical complications (by type) within 120 days follow up will be presented, in the two study arms. If there is no clear evidence of non-proportional hazards, Cox regression will be used to analyse time to death, and time to complication. Discharge destination (i.e. whether the same as pre-fracture place of residence), place of residence at 120 days (whether the same as pre-fracture place of residence) will be compared between study arms using logistic regression. For each of these outcomes, the number of events will be checked when considering adjustment factors to be included in the analyses.
Unadjusted analyses of the primary outcome and secondary outcomes will also be run. Analysis of safety outcomes (including operative complications) will be based on the per-protocol population as well as on an intention-to-treat basis.

Data Management and confidentiality

Data collection tools and source document identification

The data collection tool for this study is paper Case Report Forms (CRFs). Data are entered directly onto the CRFs and considered source documents. The CRF consists of standardised outcome measures (listed in table 1).

Data handling and record keeping

The recruitment sites store all original signed informed consent forms and copies of the CRF pages. Information on these documents is transcribed at site to a trial database. The CTU Trial Manager checks the trial database for data completeness and liaises with sites regarding any data queries.

Access to Data

Direct access is granted to authorised representatives from the Sponsor, host institution and the regulatory authorities to permit trial-related monitoring, audits and inspections in line with participant consent.

For peer review only - http://bmjopen.bmj.com/site/about/guidelines.xhtml

Data protection and patient confidentiality

The trial ID is used to identify data collected on CRFs and stored on the CTU database. Access to the CTU database is password protected and limited to those individuals necessary for quality control, audit, and analyses. The sponsor acts as the data controller for this study and will archive identifiable information for up to five years after the study has finished. Non-identifiable information will be kept in an open access archive managed by Exeter University indefinitely.

Archiving

Archiving is authorised by the Sponsor following submission of the end of trial report. The sponsor is responsible for archiving the essential documents. Exeter CTU is responsible for archiving the trial database. All essential documents will be archived for five years after completion of trial. Destruction of essential documents will require authorisation from the Sponsor.

Monitoring, audit and inspection

A Trial Monitoring Plan has been developed and agreed by the Trial Management Group (TMG), IDMC/TSC (for charter contact lead author) and CI based on the trial risk assessment. Monitoring is being conducted by the CTU Trial manager both remotely using the trial database and also with in person visits to sites. The CTU Trial Manager monitors participant enrolment, consent, eligibility, and allocation to trial groups; adherence to trial interventions and policies to protect participants, including reporting of harm and completeness, accuracy, and timeliness of data collection. Site staff are expected to assist the Trial Manager when requests for information are made or when an in person site visit is arranged.

Patient and public involvement

The premise and the primary outcome of this trial was informed by discussions with patients about key outcomes for a separate project. We continue to integrate the involvement of patients in this work, organised by patient Co-applicant AA and PPI facilitator EC. To date patients/carers with experience of hip fractures have helped develop the study design, plain English summary, patient facing documents and the qualitative interview schedule; they will also be closely involved in co-designing patient friendly dissemination materials.

ETHICS AND DISSEMINATION

Ethics

The protocol has been approved by the Yorkshire and the Humber – Bradford Leeds Research Ethics Committee (REC Reference: IRAS 258327). The trial is conducted in accordance with the study protocol, the principles of the Declaration of Helsinki, International Conference on Harmonisation of Good Clinical Practice and the Medicines for Human Use (Clinical Trials) Regulations, 2004 (17). Also in accordance with the UK Policy

For peer review only - http://bmjopen.bmj.com/site/about/guidelines.xhtml

Framework for Health and Social Care Research (18) the Mental Capacity Act 2005 (19) and the Data Protection Act 2018 (20). The trial has been adopted by the National Institute for Health Research (NIHR) Clinical Research Network and has relevant local NHS research approvals. The trial is sponsored by Royal Devon and Exeter NHS Trust, and managed by the UKCRC-registered Exeter CTU.

Amendments

Sponsor SOPs are being followed for amendments. The decision to make an amendment is made by the TMG with sponsor approval. The sponsor decides whether an amendment is substantial or non-substantial. The Exeter CTU Trial Manager submits amendments to the REC, record approvals and communicate approved amendments to sites. Amendment history will be tracked and recorded in the Trial Master File maintained by Exeter CTU.

Dissemination and impact activities

Trial progress is reported at TMG and IDMC/TSC meetings. We will follow established practice in our institution in disseminating the results of the HEMISPAIRE trial using the widest range possible of peer reviewed scientific journals, professional publications, and national academic meetings. We will present at national and international conferences. If proven to be superior, the SPAIRE technique is likely to continue at recruiting sites and also adopted in other centres around the UK. Results will be incorporated into our clinical raining programmes and we will make recommendations to regulatory bodies such as NICE. At the end of the trial we will seek input from our PPI representatives to help disseminate a lay summary of the findings to study participants. Results will be disseminated via public involvement events, patient groups, networks and media releases.

ACKNOWLEDGMENTS AND FUNDING STATEMENT

This study is funded by the National Institute for Health Research (NIHR) Research for Patient Benefit (PB-PG-0817-20039). The views expressed are those of the author(s) and not necessarily those of the NIHR or the Department of Health and Social Care.

SB and EC are supported by the National Institute for Health Research Applied Research Collaboration South West Peninsula. The views expressed in this publication are those of the authors and not necessarily those of the National Institute for Health Research or the Department of Health and Social Care.

Author declarations of competing interests

Dr Price, Dr Ball, Dr Gordon, Dr Timperley, Mr Aylward, Dr Wickins, Dr Powell, Dr Morgan-Trimmer, and Dr Cockcroft have nothing to disclose. Dr. Rhodes reports grants from NIHR RFPB, during the conduct of the study. Dr Charity reports grants from NIHR, during the conduct of the study.

For peer review only - http://bmjopen.bmj.com/site/about/guidelines.xhtml

We would like to thank all those who have contributed to this study, including; the patients and careers who have been involved in the conception and planning of this research; colleagues for their advice and support. Also the PIs, surgeon collaborators, and research teams across the NHS Trust sites that have been involved in this study: Royal Devon and Exeter, Royal Cornwall Hospitals, Northern Devon Healthcare, Somerset, Yeovil Hospital and Weston Area Health.

Author contributions

The research idea was inspired by hip hemiarthroplasty patients. All co-applicants and external advisors actively contributed with the study design. SR, SB and JC developed the protocol. AP drafted the protocol ready for publication, manages the trial, and wrote amendments for ethics submission. JC leads the trial. RW is responsible for cross-site training on outcome measures and EG is lead nurse for the lead site. SB is the trial statistician. AA leads on patient and public involvement (PPI) representation. EC provides PPI expertise. JT provides surgical research expertise. RP reviewed the manuscript: he has been a RfPB-funded trial CI and has statistical skills, design and ethics committee experience. SMT provides qualitative research expertise, supported by EC. SR provides senior trial management expertise, supported by AP as trial manager. All authors commented on the protocol and the manuscript.

For peer review only

For peer review only - http://bmjopen.bmj.com/site/about/guidelines.xhtml

REFERENCES

1. **RCP**. Royal College of Physicians. National Hip Fracture Database annual report 2017 London2016 [cited 2020. Available from: https://www.nhfd.co.uk/2017report.
2. McGilton KS, Davis AM, Naglie G, Mahomed N, Flannery J, Jaglal S, et al. Evaluation of patient-centered rehabilitation model targeting older persons with a hip fracture, including those with cognitive impairment. BMC geriatrics. 2013;13(1):136.
3. **RCP**. Royal College of Physicians. National Hip Fracture Database annual report 2016 London2016 [cited 2020. Available from: https://www.rcplondon.ac.uk/projects/outputs/national-hip-fracture-database-annual-report-2016.
4. NICE. Hip fracture: the management of hip fracture in adults (CG124) 2011 [cited 2020. Available from: https://www.nice.org.uk/guidance/cg124.
5. Kristensen TB, Vinje T, Havelin LI, Engesæter LB, Gjertsen J-E. Posterior approach compared to direct lateral approach resulted in better patient-reported outcome after hemiarthroplasty for femoral neck fracture: 20,908 patients from the Norwegian Hip Fracture Register. Acta orthopaedica. 2017;88(1):29-34.
6. Han SK, Kim YS, Kang SH. Treatment of femoral neck fractures with bipolar hemiarthroplasty using a modified minimally invasive posterior approach in patients with neurological disorders. Orthopedics. 2012;35(5):e635-e40.
7. Vaarbakken K, Steen H, Samuelsen G, Dahl HA, Leergaard TB, Nordsletten L, et al. Lengths of the external hip rotators in mobilized cadavers indicate the quadriceps coxa as a primary abductor and extensor of the flexed hip. Clinical Biomechanics. 2014;29(7):794-802.
8. Fullam J, Theodosi PG, Charity J, Goodwin VA. A scoping review comparing two common surgical approaches to the hip for hemiarthroplasty. BMC surgery. 2019;19(1):32.
9. Parker MJ. Lateral versus posterior approach for insertion of hemiarthroplasties for hip fractures: A randomised trial of 216 patients. Injury. 2015;46(6):1023-7.
10. Braun V, Clarke V. Using thematic analysis in psychology. Qualitative research in psychology. 2006;3(2):77-101.
11. QSR International Pty Ltd. NVivo qualitative data analysis software, version 12. 2018.
12. Dawson J, Fitzpatrick R, Carr A, Murray D. Questionnaire on the perceptions of patients about total hip replacement. The Journal of bone and joint surgery British volume. 1996;78(2):185-90.
13. Murray D, Fitzpatrick R, Rogers K, Pandit H, Beard D, Carr A, et al. The use of the Oxford hip and knee scores. The Journal of bone and joint surgery British volume. 2007;89(8):1010-4.
14. Beard DJ, Harris K, Dawson J, Doll H, Murray DW, Carr AJ, et al. Meaningful changes for the Oxford hip and knee scores after joint replacement surgery. Journal of clinical epidemiology. 2015;68(1):73-9.
15. Schulz KF, Altman DG, Moher D. CONSORT 2010 statement: updated guidelines for reporting parallel group randomised trials. BMC medicine. 2010;8(1):18.
16. de Morton NA, Harding KE, Taylor NF, Harrison G. Validity of the de Morton Mobility Index (DEMMI) for measuring the mobility of patients with hip fracture during rehabilitation. Disability and rehabilitation. 2013;35(4):325-33.
17. Statutory Instrument No 1031. Medicines for Human Use (Clinical Trials) Regulations 2004. London: The Stationery Office; 2004.

For peer review only - http://bmjopen.bmj.com/site/about/guidelines.xhtml

18. NHS Health Research Authority. UK policy framework for health and social care research. London: Health Research Authority 2017.
19. Statutory Instrument. Mental Capacity Act 2005. London: The Stationery Office; 2005.
20. Statutory Instrument. Data Protection Act 2018. London: The Stationery Office; 2018.

Figure legend

Figure 1: Flow chart of trial design, with anticipated numbers of participants

For peer review only

For peer review only - http://bmjopen.bmj.com/site/about/guidelines.xhtml

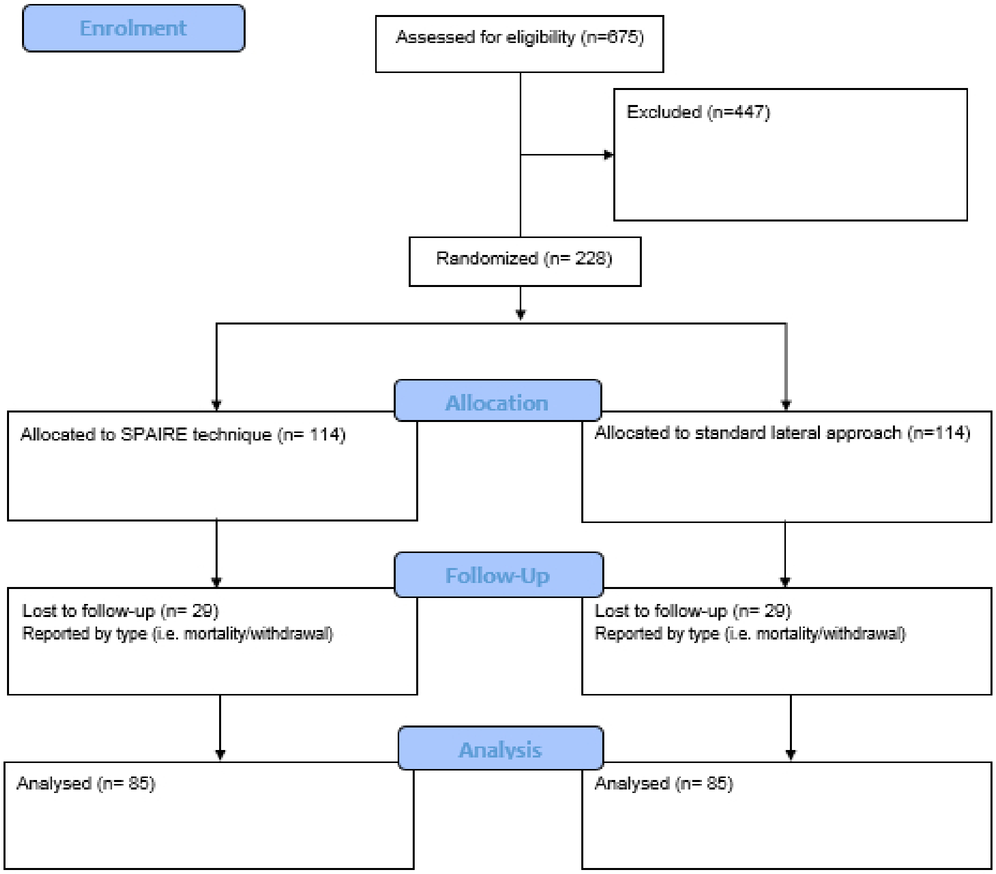

Figure 1: Flow chart of trial design, with anticipated numbers of participants

National Institute for Health Research

Participant consent form

The HemiSPAIRE study:
The effects of a modified muscle sparing posterior technique (SPAIRE) in hip hemiarthroplasty for displaced intracapsular fractures on post-operative function compared to a standard lateral approach; a randomised controlled trial.

CONSENT FORM

Please see that the consent form **is in two parts, you do not have to sign both parts:**

Part 1 on Page 1:
This is the main consent for your general participation in the study and if you agree to taking part.

Part 2 on Page 2 is optional; you can choose if you wish to take part.
This is about whether you would agree to being interviewed about your experiences of recovery from surgery. It also includes a similar section about data collection.

PART 1: MAIN STUDY CONSENT FORM

Site Details:

	Please initial box
1. I confirm that I have read and understand the information sheet dated version for the above study and have had the opportunity to consider the information, ask questions and have had these answered satisfactorily.	
2. I agree to my usual care giver being informed of my participation in this study and updated with information from this study relevant to my medical care.	
3. I understand that my participation is voluntary and that I am free to withdraw at any time, without giving any reason and without my medical care or legal rights being affected.	
4. I understand that relevant sections of my medical notes and data collected during the study may be looked at by individuals, from regulatory authorities or from the NHS Trust, where it is relevant to my taking part in the research. I give permission for these individuals to have access to my records.	
5. I understand that identifiable data already collected as part of the research study can be retained for up to 5 years, even if I decide to withdraw from the study and that it will only be used for this study.	
6. I understand that the anonymised data collected from the study will be retained in secure storage during the study and after the study will be stored in a repository.	
7. I agree to my General Practitioner being informed of my participation in the study.	
8. I agree to take part in the above study.	

When you have initialled the boxes above, please complete below including the date yourself.

Name of Participant *(BLOCK CAPITALS)* **Date** **Signature**

I have explained the study to the above patient and he/she has indicated his/her willingness to take part in the study.

Name of Researcher *(BLOCK CAPITALS)* **Date** **Signature**

You will be given a copy of this consent form and the information sheet to keep. A copy of the consent form will be filed in your patient notes, one will be filed with the study records and one may be sent to the Research Sponsor (Royal Devon and Exeter).

For peer review only - http://bmjopen.bmj.com/site/about/guidelines.xhtml

NHS National Institute for Health Research

Participant consent form

The HemiSPAIRE study:
The effects of a modified muscle sparing posterior technique (SPAIRE) in hip hemiarthroplasty for displaced intracapsular fractures on post-operative function compared to a standard lateral approach; a randomised controlled trial.

The HemiSPAIRE study: The effects of a modified muscle sparing posterior technique (SPAIRE) in hip hemiarthroplasty for displaced intracapsular fractures on post-operative function compared to a standard lateral approach; a randomised controlled trial.

PART 2: OPTIONAL CONSENT FORM

Part 2: This section is optional; you can choose if you wish to take part or not and it will not affect your participation in the main part of the study.

This consent form is about whether you would agree to:

- *Being interviewed about your experiences of recovery from surgery?*

Please only initial the boxes that you wish to consent to, thank you.

Site Details

	**Please only initial the boxes that apply**
1. I am willing to be interviewed about my experiences of recovery from hip fracture surgery and for this interview to be audio recorded for research purposes	
2. I agree to the data collected for this additional part of the above study being retained for up to 5 years.	
3. I agree to my data from this study being shared with other health researchers after my personal identifying information has been removed. I understand that it will only be used towards improving health outcomes by assessing the types of treatment that I have agreed to participate in for the main study.	
4. I understand that quotes from my interview may be used in study publications such as journal articles, and that these quotes will not contain any information that would enable someone to identify me.	
5. I agree to this additional part of the above study	

Name of Participant *(BLOCK CAPITALS)* **Date** **Signature**

I have explained the additional part of study to the above patient and he/she has indicated which parts apply.

Name of Researcher *(BLOCK CAPITALS)* **Date** **Signature**

For peer review only - http://bmjopen.bmj.com/site/about/guidelines.xhtml

Consultee declaration form

National Institute for Health Research

The HemiSPAIRE study:
The effects of a modified muscle sparing posterior technique (SPAIRE) in hip hemiarthroplasty for displaced intracapsular fractures on post-operative function compared to a standard lateral approach; a randomised controlled trial.

CONSULTEE DECLARATION FORM

Site Details:

	Please initial box
1. I [*name of consultee (see below)]* have been consulted about [*name of potential participant* ______________ ______________]'s participation in this research project. I have had the opportunity to ask questions about the study and understand what is involved.	
2. In my opinion he/she would have no objection to taking part in the above study.	
3. I understand that his/her participation is voluntary and that they are free to withdraw at any time, without giving any reason and without their medical care or legal rights being affected.	
4. I understand that relevant sections of his/her medical notes and data collected during the study may be looked at by individuals from the HemiSPAIRE project team, and from regulatory authorities where it is relevant to his/her taking part in the research. I give permission for these individuals to have access to their records.	
5. I understand that the information collected about him/her may be published and shared anonymously with other researchers. The information may be used to support other research in the future.	
6. I understand that the anonymised data collected from the study will be retained in secure storage during the study and after the study will be stored in a repository.	
7. I agree to his/her General Practitioner being informed of their participation in the study.	

When you have initialled the boxes above, please complete below including the date yourself.

Name of Consultee *(BLOCK CAPITALS)* **Date** **Signature**

Relationship to Patient *(BLOCK CAPITALS)*

I have explained the study to the above consultee and he/she has indicated his/her willingness to consent to the patient taking part in the study.

Name of Researcher *(BLOCK CAPITALS)* **Date** **Signature**

You will be given a copy of this declaration form and the information sheet to keep. A copy of the declaration form will be filed in the patient notes, one will be filed with the study records and one may be sent to the Research Sponsor (Royal Devon and Exeter).

For peer review only - http://bmjopen.bmj.com/site/about/guidelines.xhtml

SPIRIT
STANDARD PROTOCOL ITEMS: RECOMMENDATIONS FOR INTERVENTIONAL TRIALS

SPIRIT 2013 Checklist for the HemiSPAIRE study: Recommended items to address in a clinical trial protocol and related documents*

Section/item	Item No	Description	Addressed on page number
**Administrative information**			
Title	1	Descriptive title identifying the study design, population, interventions, and, if applicable, trial acronym	1
Trial registration	2a	Trial identifier and registry name. If not yet registered, name of intended registry	2
	2b	All items from the World Health Organization Trial Registration Data Set	Yes, clinicaltrials.gov
Protocol version	3	Date and version identifier	1
Funding	4	Sources and types of financial, material, and other support	14,15
Roles and responsibilities	5a	Names, affiliations, and roles of protocol contributors	1&14
	5b	Name and contact information for the trial sponsor	1
	5c	Role of study sponsor and funders, if any, in study design; collection, management, analysis, and interpretation of data; writing of the report; and the decision to submit the report for publication, including whether they will have ultimate authority over any of these activities	14
	5d	Composition, roles, and responsibilities of the coordinating centre, steering committee, endpoint adjudication committee, data management team, and other individuals or groups overseeing the trial, if applicable (see Item 21a for data monitoring committee)	12,13,14

For peer review only

**Introduction**			
Background and rationale	6a	Description of research question and justification for undertaking the trial, including summary of relevant studies (published and unpublished) examining benefits and harms for each intervention	4, 5
	6b	Explanation for choice of comparators	5
Objectives	7	Specific objectives or hypotheses	5, 6
Trial design	8	Description of trial design including type of trial (eg, parallel group, crossover, factorial, single group), allocation ratio, and framework (eg, superiority, equivalence, noninferiority, exploratory)	5, 6
**Methods: Participants, interventions, and outcomes**			
Study setting	9	Description of study settings (eg, community clinic, academic hospital) and list of countries where data will be collected. Reference to where list of study sites can be obtained	1, 6
Eligibility criteria	10	Inclusion and exclusion criteria for participants. If applicable, eligibility criteria for study centres and individuals who will perform the interventions (eg, surgeons, psychotherapists)	7
Interventions	11a	Interventions for each group with sufficient detail to allow replication, including how and when they will be administered	8
	11b	Criteria for discontinuing or modifying allocated interventions for a given trial participant (eg, drug dose change in response to harms, participant request, or improving/worsening disease)	10
	11c	Strategies to improve adherence to intervention protocols, and any procedures for monitoring adherence (eg, drug tablet return, laboratory tests)	10, 11
	11d	Relevant concomitant care and interventions that are permitted or prohibited during the trial	10, 11
Outcomes	12	Primary, secondary, and other outcomes, including the specific measurement variable (eg, systolic blood pressure), analysis metric (eg, change from baseline, final value, time to event), method of aggregation (eg, median, proportion), and time point for each outcome. Explanation of the clinical relevance of chosen efficacy and harm outcomes is strongly recommended	Table 1, (9)
Participant timeline	13	Time schedule of enrolment, interventions (including any run-ins and washouts), assessments, and visits for participants. A schematic diagram is highly recommended (see Figure)	Table 1 (9) & Figure 1 (6), 7, 8

For peer review only - http://bmjopen.bmj.com/site/about/guidelines.xhtml

Sample size	14	Estimated number of participants needed to achieve study objectives and how it was determined, including clinical and statistical assumptions supporting any sample size calculations	10,11
Recruitment	15	Strategies for achieving adequate participant enrolment to reach target sample size	11

Methods: Assignment of interventions (for controlled trials)

Allocation:			
Sequence generation	16a	Method of generating the allocation sequence (eg, computer-generated random numbers), and list of any factors for stratification. To reduce predictability of a random sequence, details of any planned restriction (eg, blocking) should be provided in a separate document that is unavailable to those who enrol participants or assign interventions	7, 8
Allocation concealment mechanism	16b	Mechanism of implementing the allocation sequence (eg, central telephone; sequentially numbered, opaque, sealed envelopes), describing any steps to conceal the sequence until interventions are assigned	11
Implementation	16c	Who will generate the allocation sequence, who will enrol participants, and who will assign participants to interventions	7,8,11,12
Blinding (masking)	17a	Who will be blinded after assignment to interventions (eg, trial participants, care providers, outcome assessors, data analysts), and how	8
	17b	If blinded, circumstances under which unblinding is permissible, and procedure for revealing a participant's allocated intervention during the trial	8,10, 12

Methods: Data collection, management, and analysis

Data collection methods	18a	Plans for assessment and collection of outcome, baseline, and other trial data, including any related processes to promote data quality (eg, duplicate measurements, training of assessors) and a description of study instruments (eg, questionnaires, laboratory tests) along with their reliability and validity, if known. Reference to where data collection forms can be found, if not in the protocol	Table 1 (9) 11, 12, 13
	18b	Plans to promote participant retention and complete follow-up, including list of any outcome data to be collected for participants who discontinue or deviate from intervention protocols	Table 1 (9), 12, 13

Data management	19	Plans for data entry, coding, security, and storage, including any related processes to promote data quality (eg, double data entry; range checks for data values). Reference to where details of data management procedures can be found, if not in the protocol	12,13
Statistical methods	20a	Statistical methods for analysing primary and secondary outcomes. Reference to where other details of the statistical analysis plan can be found, if not in the protocol	11, 12
	20b	Methods for any additional analyses (eg, subgroup and adjusted analyses)	11, 12
	20c	Definition of analysis population relating to protocol non-adherence (eg, as randomised analysis), and any statistical methods to handle missing data (eg, multiple imputation)	11, 12
**Methods: Monitoring**			
Data monitoring	21a	Composition of data monitoring committee (DMC); summary of its role and reporting structure; statement of whether it is independent from the sponsor and competing interests; and reference to where further details about its charter can be found, if not in the protocol. Alternatively, an explanation of why a DMC is not needed	10, 11, 12
	21b	Description of any interim analyses and stopping guidelines, including who will have access to these interim results and make the final decision to terminate the trial	11
Harms	22	Plans for collecting, assessing, reporting, and managing solicited and spontaneously reported adverse events and other unintended effects of trial interventions or trial conduct	10
Auditing	23	Frequency and procedures for auditing trial conduct, if any, and whether the process will be independent from investigators and the sponsor	11
**Ethics and dissemination**			
Research ethics approval	24	Plans for seeking research ethics committee/institutional review board (REC/IRB) approval	1, 13
Protocol amendments	25	Plans for communicating important protocol modifications (eg, changes to eligibility criteria, outcomes, analyses) to relevant parties (eg, investigators, REC/IRBs, trial participants, trial registries, journals, regulators)	12

For peer review only - http://bmjopen.bmj.com/site/about/guidelines.xhtml

Consent or assent	26a	Who will obtain informed consent or assent from potential trial participants or authorised surrogates, and how (see Item 32)	7
	26b	Additional consent provisions for collection and use of participant data and biological specimens in ancillary studies, if applicable	N/A
Confidentiality	27	How personal information about potential and enrolled participants will be collected, shared, and maintained in order to protect confidentiality before, during, and after the trial	12, 13
Declaration of interests	28	Financial and other competing interests for principal investigators for the overall trial and each study site	14
Access to data	29	Statement of who will have access to the final trial dataset, and disclosure of contractual agreements that limit such access for investigators	14
Ancillary and post-trial care	30	Provisions, if any, for ancillary and post-trial care, and for compensation to those who suffer harm from trial participation	N/A
Dissemination policy	31a	Plans for investigators and sponsor to communicate trial results to participants, healthcare professionals, the public, and other relevant groups (eg, via publication, reporting in results databases, or other data sharing arrangements), including any publication restrictions	13
	31b	Authorship eligibility guidelines and any intended use of professional writers	14
	31c	Plans, if any, for granting public access to the full protocol, participant-level dataset, and statistical code	
**Appendices**			
Informed consent materials	32	Model consent form and other related documentation given to participants and authorised surrogates	Consent and Declaration forms available as supplementary material. P7~~Available on request~~

Biological specimens	33	Plans for collection, laboratory evaluation, and storage of biological specimens for genetic or molecular analysis in the current trial and for future use in ancillary studies, if applicable	n/a

*It is strongly recommended that this checklist be read in conjunction with the SPIRIT 2013 Explanation & Elaboration for important clarification on the items. Amendments to the protocol should be tracked and dated. The SPIRIT checklist is copyrighted by the SPIRIT Group under the Creative Commons "Attribution-NonCommercial-NoDerivs 3.0 Unported" license.

a) WHO Trial Registration Data Set (Version 1.2.1)

The minimum amount of trial information that must appear in a register in order for a given trial to be considered fully registered. There are currently 20 items in the WHO Trial Registration Data Set. It is sometimes referred to as the TRDS.

- **Primary Registry and Trial Identifying Number**
  Name of Primary Registry, and the unique ID number assigned by the Primary Registry to this trial.

- **Date of Registration in Primary Registry**
  Date when trial was officially registered in the Primary Registry.

- **Secondary Identifying Numbers**
  Other identifiers besides the Trial Identifying Number allocated by the Primary Registry, if any. These include:
  - The Universal Trial Number (UTN)
  - Identifiers assigned by the sponsor (record Sponsor name and Sponsor-issued trial number (e.g. protocol number))
  - Other trial registration numbers issued by other Registries (both Primary and Partner Registries in the WHO Registry Network, and other registries)
  - Identifiers issued by funding bodies, collaborative research groups, regulatory authorities, ethics committees / institutional review boards, etc.

  All secondary identifiers will have 2 elements: an identifier for the issuing authority (e.g. NCT, ISRCTN, ACTRN) plus a number.

  There is no limit to the number of secondary identifiers that can be provided.

- **Source(s) of Monetary or Material Support**
  Major source(s) of monetary or material support for the trial (e.g. funding agency, foundation, company, institution).

For peer review only - http://bmjopen.bmj.com/site/about/guidelines.xhtml

- **Primary Sponsor**
The individual, organization, group or other legal entity which takes responsibility for initiating, managing and/or financing a study. The Primary Sponsor is responsible for ensuring that the trial is properly registered. The Primary Sponsor may or may not be the main funder.

- **Secondary Sponsor(s)**
Additional individuals, organizations or other legal persons, if any, that have agreed with the primary sponsor to take on responsibilities of sponsorship.

  A secondary sponsor may have agreed to:
  - take on all the responsibilities of sponsorship jointly with the primary sponsor; or
  - form a group with the Primary Sponsor in which the responsibilities of sponsorship are allocated among the members of the group; or
  - act as the Primary Sponsor's legal representative in relation to some or all of the trial sites.

- **Contact for Public Queries**
Email address, telephone number and postal address of the contact who will respond to general queries, including information about current recruitment status.

- **Contact for Scientific Queries**
There must be clearly assigned responsibility for scientific leadership to a named Principal Investigator. The PI may delegate responsibility for dealing with scientific enquiries to a scientific contact for the trial. This scientific contact will be listed in addition to the PI.

  The contact for scientific queries must therefore include:
  - Name and title, email address, telephone number, postal address and affiliation of the Principal Investigator, and;
  - Email address, telephone number, postal address and affiliation of the contact for scientific queries about the trial (if applicable). The details for the scientific contact may be generic (that is, there does not need to be a named individual): e.g. a generic email address for research team members qualified to answer scientific queries.

- **Public Title**
Title intended for the lay public in easily understood language.

- **Scientific Title**
Scientific title of the study as it appears in the protocol submitted for funding and ethical review. Include trial acronym if available.

- **Countries of Recruitment**
The countries from which participants will be, are intended to be, or have been recruited at the time of registration.

- **Health Condition(s) or Problem(s) Studied**
Primary health condition(s) or problem(s) studied (e.g., depression, breast cancer, medication error).

For peer review only

If the study is conducted in healthy human volunteers belonging to the target population of the intervention (e.g. preventive or screening interventions), enter the particular health condition(s) or problem(s) being prevented.

- **Intervention(s)**
  For each arm of the trial record a brief intervention name plus an intervention description.

  Intervention Name: For drugs use generic name; for other types of interventions provide a brief descriptive name.
    - For investigational new drugs that do not yet have a generic name, a chemical name, company code or serial number may be used on a temporary basis. As soon as the generic name has been established, update the associated registered records accordingly.
    - For non-drug intervention types, provide an intervention name with sufficient detail so that it can be distinguished from other similar interventions.

  Intervention Description: Must be sufficiently detailed for it to be possible to distinguish between the arms of a study (e.g. comparison of different dosages of drug) and/or among similar interventions (e.g. comparison of multiple implantable cardiac defibrillators). For example, interventions involving drugs may include dosage form, dosage, frequency and duration.

  If the intervention is one or more drugs then use the International Non-Proprietary Name for each drug if possible (not brand/trade names). For an unregistered drug, the generic name, chemical name, or company serial number is acceptable.

  If the intervention consists of several separate treatments, list them all in one line separated by commas (e.g. "low-fat diet, exercise").

  For controlled trials, the identity of the control arm should be clear. The control intervention(s) is/are the interventions against which the study intervention is evaluated (e.g. placebo, no treatment, active control). If an active control is used, be sure to enter in the name(s) of that intervention, or enter "placebo" or "no treatment" as applicable. For each intervention, describe other intervention details as applicable (dose, duration, mode of administration, etc).

- **Key Inclusion and Exclusion Criteria**
  Inclusion and exclusion criteria for participant selection, including age and sex. Other selection criteria may relate to clinical diagnosis and co-morbid conditions; exclusion criteria are often used to ensure patient safety.

  If the study is conducted in healthy human volunteers not belonging to the target population (e.g. a preliminary safety study), enter "healthy human volunteer".

- **Study Type**
  Study type consists of:

For peer review only - http://bmjopen.bmj.com/site/about/guidelines.xhtml

For peer review only

- Type of study (interventional or observational)
- Study design including:
  - Method of allocation (randomized/non-randomized)
  - Masking (is masking used and, if so, who is masked)
  - Assignment (single arm, parallel, crossover or factorial)
  - Purpose
- Phase (if applicable)

For randomized trials: the allocation concealment mechanism and sequence generation will be documented.

- **Date of First Enrollment**
  Anticipated or actual date of enrolment of the first participant.

- **Target Sample Size**
  Number of participants that this trial plans to enrol in total.

- **Recruitment Status**
  Recruitment status of this trial:
  - Pending: participants are not yet being recruited or enrolled at any site
  - Recruiting: participants are currently being recruited and enrolled
  - Suspended: there is a temporary halt in recruitment and enrolment
  - Complete: participants are no longer being recruited or enrolled
  - Other

- **Primary Outcome(s)**
  Outcomes are events, variables, or experiences that are measured because it is believed that they may be influenced by the intervention.

  The Primary Outcome should be the outcome used in sample size calculations, or the main outcome(s) used to determine the effects of the intervention(s). Most trials should have only one primary outcome.

  For each primary outcome provide:
  - The name of the outcome (do not use abbreviations)
  - The metric or method of measurement used (be as specific as possible)
  - The timepoint(s) of primary interest

For peer review only

For peer review only - http://bmjopen.bmj.com/site/about/guidelines.xhtml

Example:
Outcome Name: Depression
Metric/method of measurement: Beck Depression Score
Timepoint: 18 weeks following end of treatment

- **Key Secondary Outcomes**
  Secondary outcomes are outcomes which are of secondary interest or that are measured at timepoints of secondary interest. A secondary outcome may involve the same event, variable, or experience as the primary outcome, but measured at timepoints other than those of primary interest.

  As for primary outcomes, for each secondary outcome provide:
    - The name of the outcome (do not use abbreviations)
    - The metric or method of measurement used (be as specific as possible)
    - The timepoint(s) of interest

For peer review only
